# Supplementary figures and images for: Enterococcus faecalis alters endo-lysosomal trafficking to replicate and persist within mammalian cells
Source: PLoS Pathog. 2022 Apr 7;18(4):e1010434. doi: 10.1371/journal.ppat.1010434 (PMC9017951; doi:10.1371/journal.ppat.1010434)

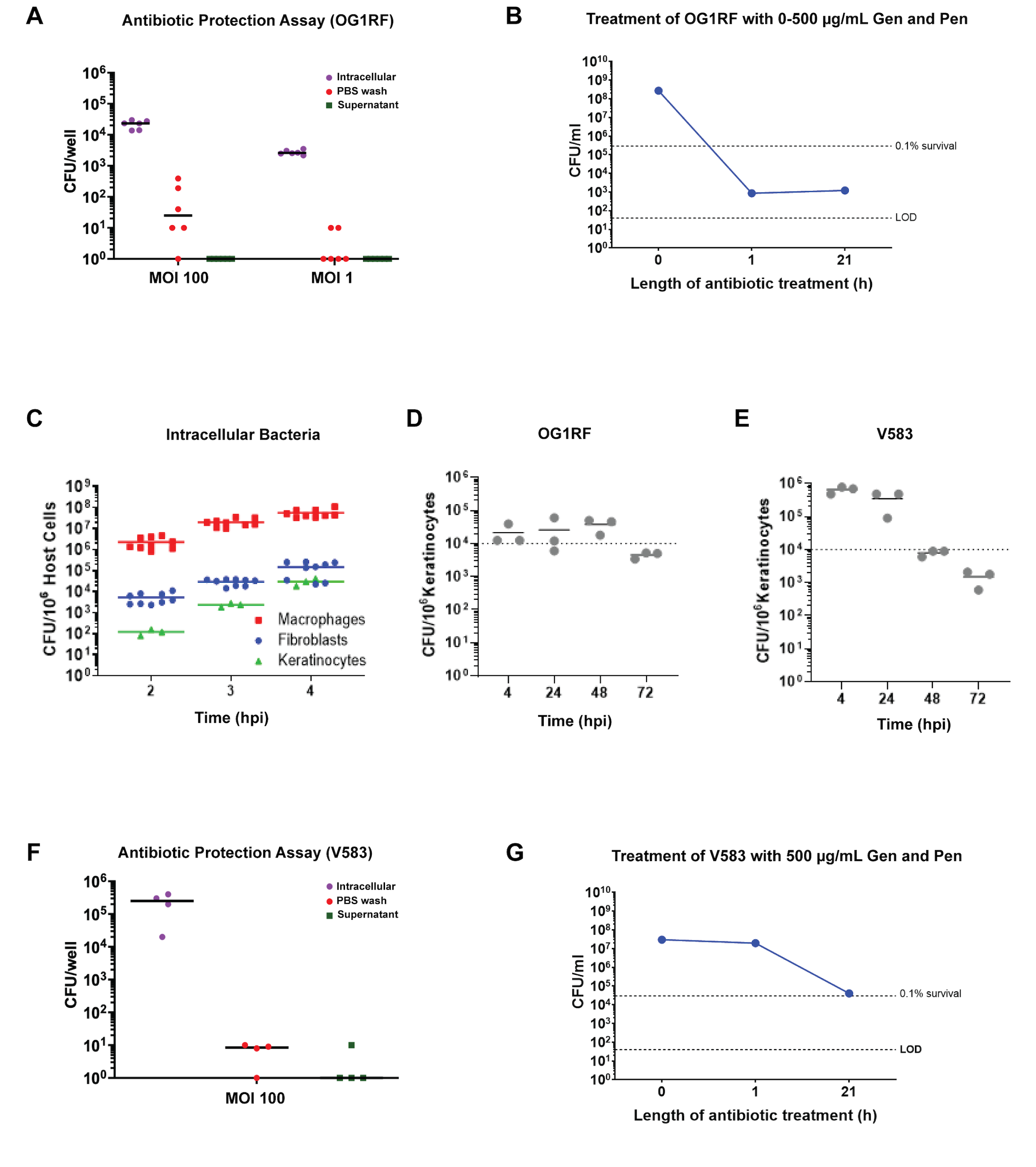

Supplement: S1 Fig — Intracellular E. faecalis is not cell type specific and persists for up to 72 hpi. (A,F) Enumeration of CFU for OG1RF was performed at different steps of the antibiotic protection assay on HaCaT cells to determine the number of bacteria found intracellularly, compared to the number of bacteria found in the supernatant and final PBS wash after antibiotic treatment. (A) reflects 3 h of infection followed by 1 h of antibiotic treatment. CFU in (F) were enumerated CFU after 3 h of infection and 21 h of antibiotic treatment when optimal killing for strain V583 is achieved. (B,G) Enumeration of CFU after antibiotic killing in planktonic cultures of OG1RF and V583 in DMEM + 10% FBS. Planktonic cultures were grown for 3 h at an inoculum size equivalent to MOI 100 from the antibiotic protection assay prior to addition of antibiotics. Cultures were incubated in the presence of antibiotics for either 1 h or 21 h at 37°C with 5% CO2. Bacteria were pelleted and resuspended in sterile 1×PBS to remove residual antibiotics before CFU enumeration. For OG1RF at 1 h and 21 h post antibiotic treatment, and V583 at 21 h post antibiotic treatment, zero CFU counts were observed when bacteria were not resuspended in 1×PBS before CFU enumeration. (C) Solid lines indicate the mean CFU at 2–4 hpi at MOI 100 from at least 3 independent experiments. (D,E) HaCaTs were infected with E. faecalis OG1RF and V583 at MOI 100 for 3 h, followed by treatment with gentamicin and penicillin for 1, 21, 45, 69 h before lysis to obtain the intracellular population. Solid lines indicate the mean CFU from at least 2 independent experiments. Dashed lines serve as point of reference for 104 CFU, for easy visualization of the comparative increase in V583 CFU. (TIF) [file ppat.1010434.s001.tif]

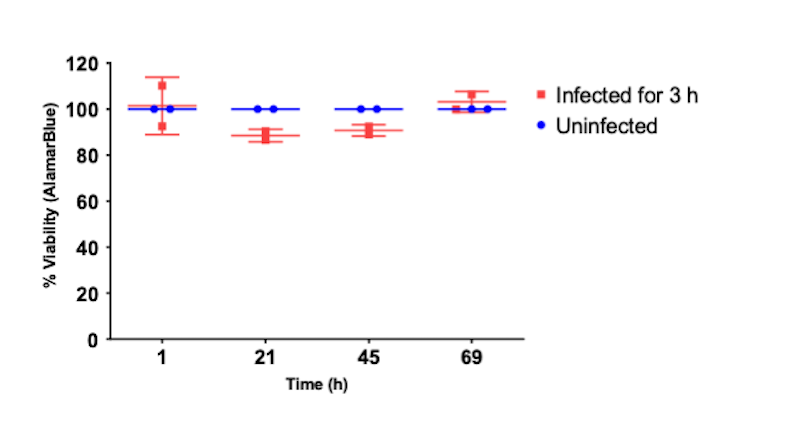

Supplement: S2 Fig — Viability of HaCaT cells upon infection with E. faecalis OG1RF. HaCaT cells were infected with MOI 100 of E. faecalis OG1RF for 3 h and incubated with 500 μg/ml of gentamicin and penicillin up to 69 hpi and subsequently assessed for viability using the AlamarBlue cell viability reagent. (TIFF) [file ppat.1010434.s002.tiff]

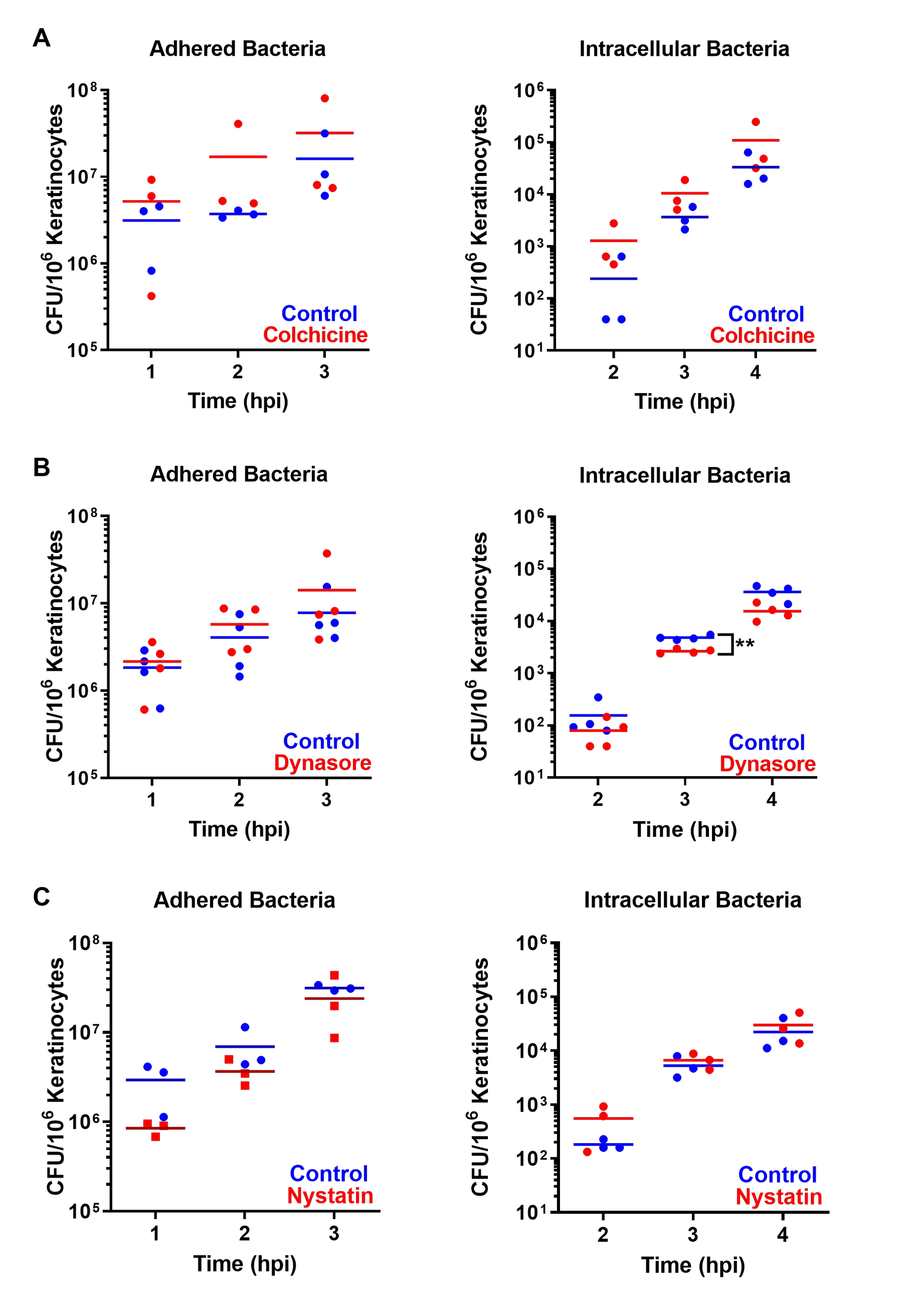

Supplement: S3 Fig — E. faecalis entry into keratinocytes is not dependent on microtubule polymerization, clathrin- and caveolae-mediated endocytosis. Keratinocytes were pre-treated with (A) microtubule inhibitor colchicine (10 μg/ml), (B) dynasore, an inhibitor of the large GTPase dynamin that is important for the formation of clathrin-coated vesicles (80) (25 μg/ml), or (C) nystatin, which selectively affects caveolae-mediated endocytosis by binding sterols, causing caveolae and cholesterol disassembly in the plasma membrane (81, 82) (25 μg/ml). Cells were pre-treated with compounds for 0.5 h and then infected with E. faecalis at MOI 100 for 1, 2, or 3 h. For enumeration of intracellular CFU, each infection period was followed by 1 h antibiotic treatment, for a total of 2, 3 or 4 hpi. Adherent or intracellular bacteria were enumerated at the indicated time points (only significant differences are indicated). Solid lines indicate the mean for each data set of at least 3 independent experiments. **p<0.01 2 way ANOVA, Sidak’s multiple comparisons test. (TIF) [file ppat.1010434.s003.tif]

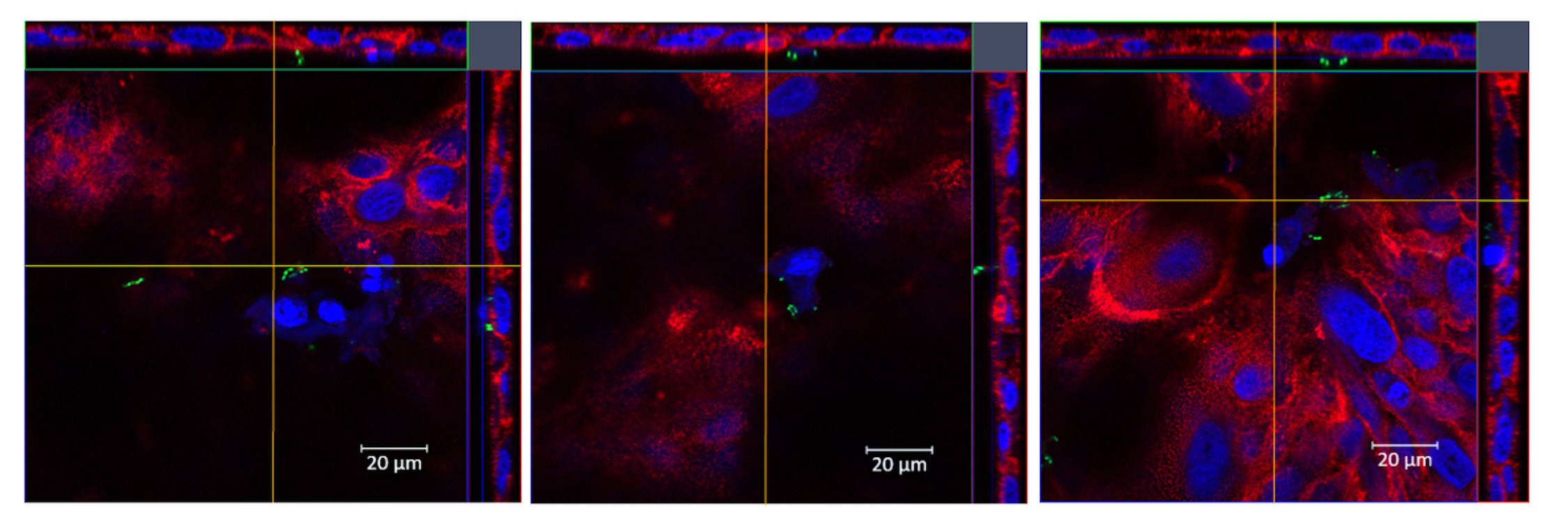

Supplement: S4 Fig — E. faecalis at the periphery of keratinocytes at 24 hpi. CLSM representative images of infected keratinocytes with condensed nuclei following 3 h of infection and 21 h of incubation in antibiotic laced media. Blue, dsDNA stained with Hoechst 33342; green, E-GFP E. faecalis; red, F-actin. Data shown are representative of at least 3 independent experiments. (TIFF) [file ppat.1010434.s004.tiff]

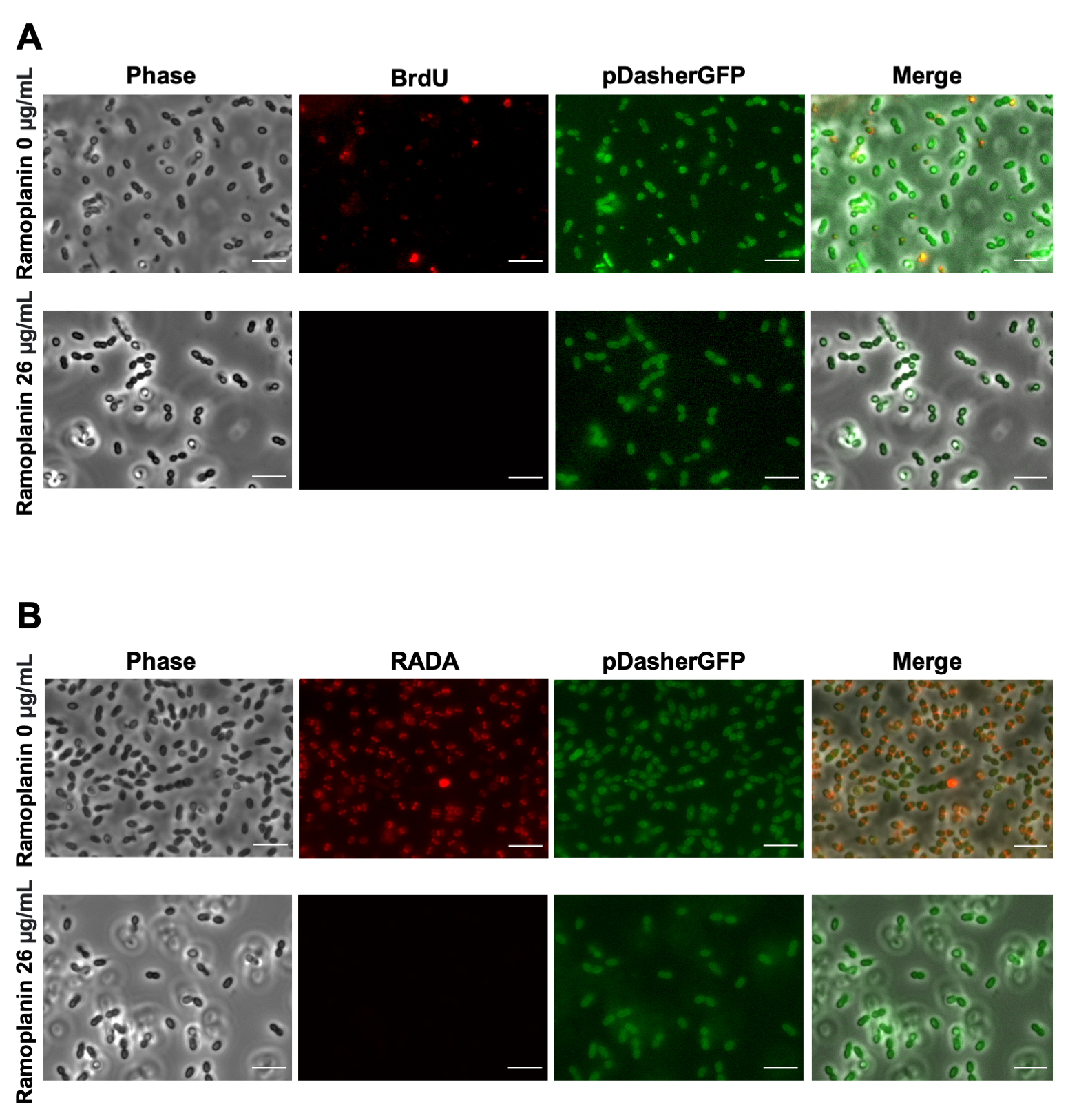

Supplement: S5 Fig — Non-replicating E. faecalis do not incorporate BrdU nor RADA. Fluorescent E. faecalis (pDasherGFP) was treated with the antibiotic ramoplanin to halt replication. (A) BrdU and (B) RADA labelling of bacteria in presence or absence of 26 μg/ml ramoplanin for 1 h. Scale bar: 2 μm. (TIFF) [file ppat.1010434.s005.tiff]

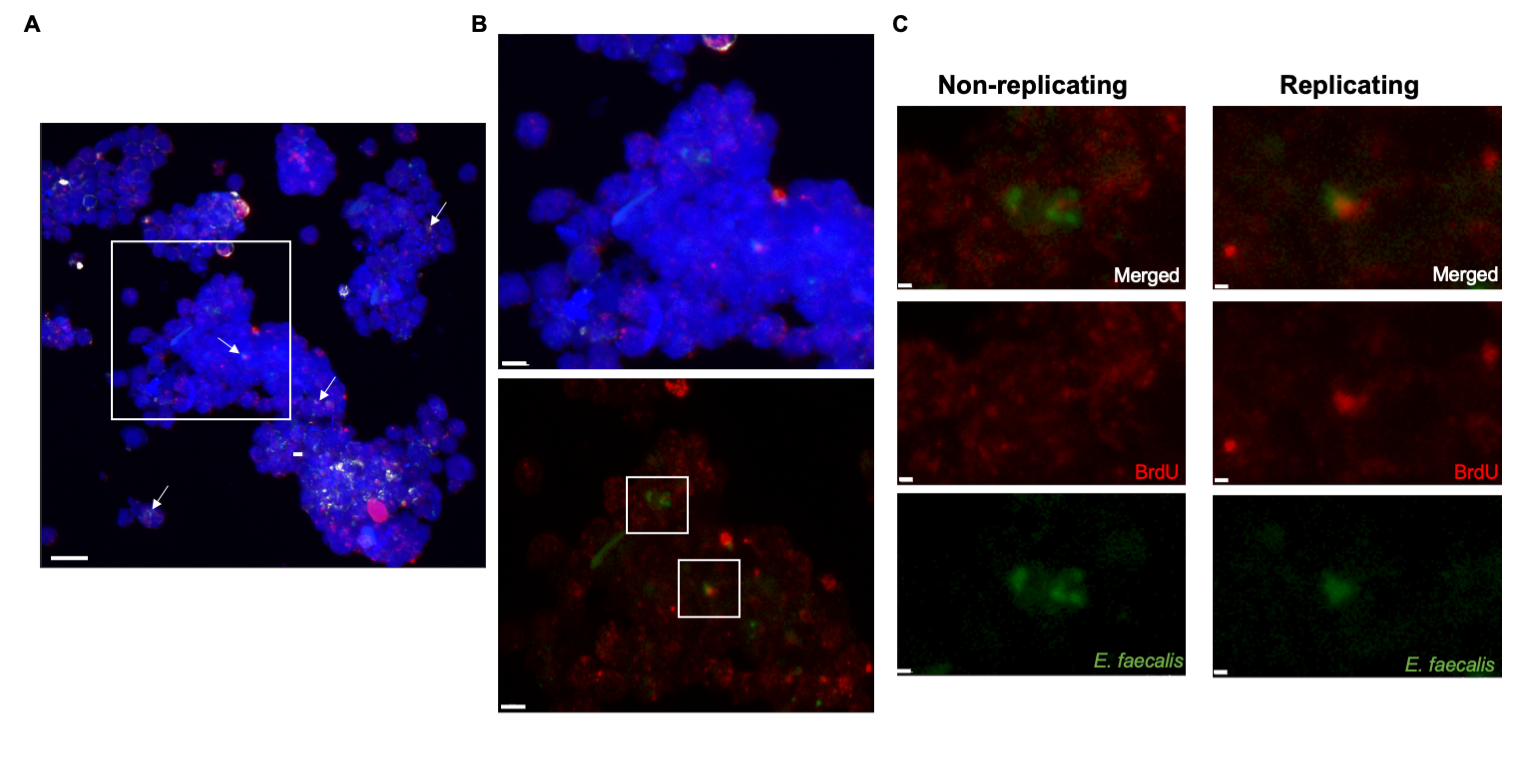

Supplement: S6 Fig — Replicating and non-replicating intracellular E. faecalis in ex vivo cells isolated from infected wounds. (A) CLSM view of ex vivo murine wound tissue cells following infection and BrdU treatment. Left panel shows multiple examples of potentially replicating E. faecalis clusters, indicated with white arrows. Scale bar: 10 μm. (B) Enlarged area within white box in (A) on the top, and the same area with the Hoechst channel removed for clear viewing of the other markers on the bottom. The marked areas with white squares show CD45-negative E. faecalis containing cells. Scale bar: 2 μm. (C) Enlarged areas within white boxes in B show examples of non-replicating and replicating E. faecalis. Blue, dsDNA stained with Hoechst 33342; green, E. faecalis; red, BrdU; white, CD45. Images are representative of 3 independent experiments. (TIFF) [file ppat.1010434.s006.tiff]

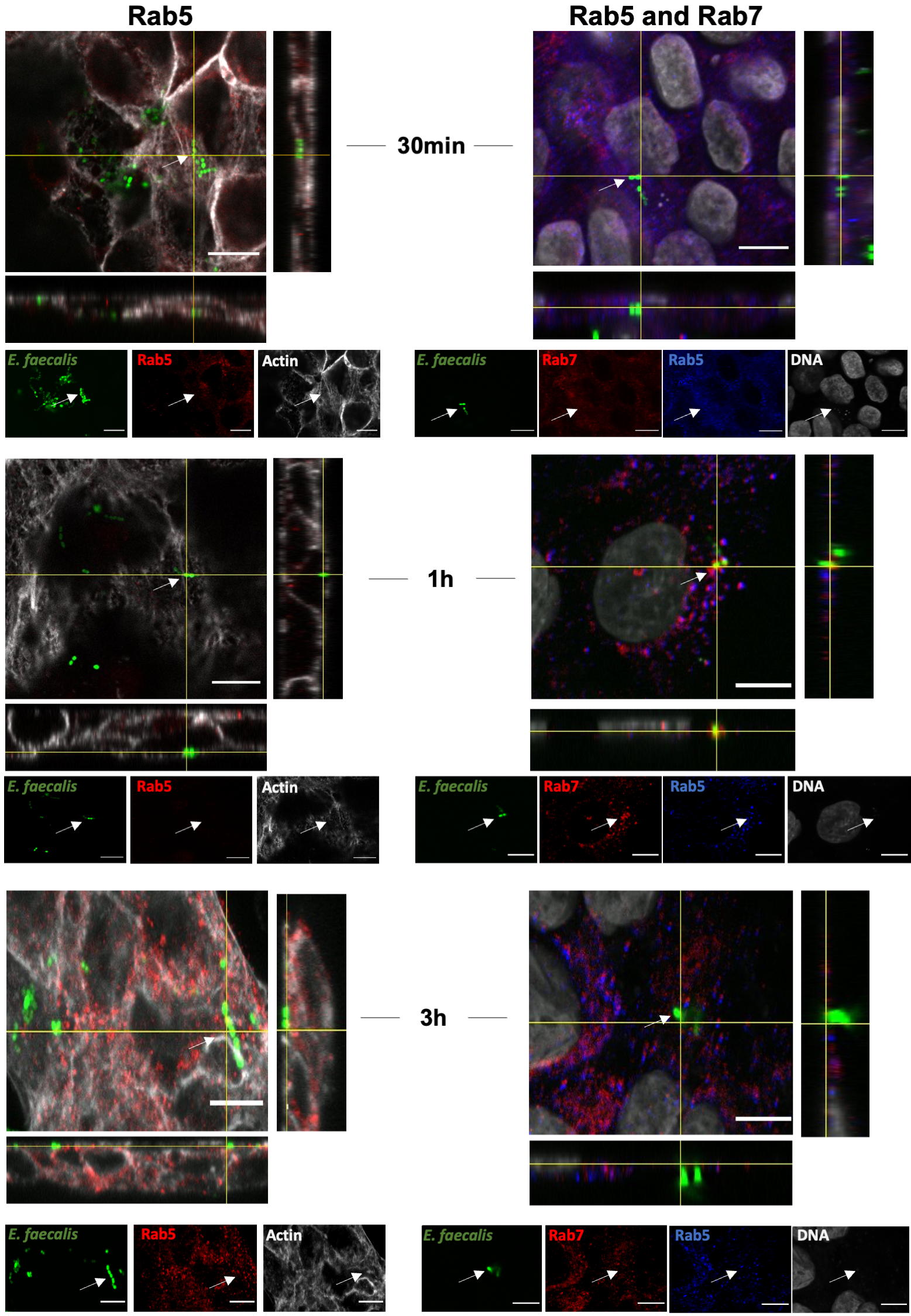

Supplement: S7 Fig — Most Rab5 and Rab7 compartments in E. faecalis infected keratinocytes do not colocalize with E. faecalis-containing compartment. CLSM Orthogonal views and individual channels of E. faecalis within keratinocytes labelled with antibodies against Rab5 (alone, left panels) or together with Rab7 (right panels) at 30 min, 1 h and 3 hpi. Left Panels: White, F-actin; green, E. faecalis (pDasherGFP); red, Rab5. Right panels: white, dsDNA stained with Hoechst 33342; green, E. faecalis (pDasherGFP); red, Rab7; blue, Rab5. Images are representative of 3 independent experiments. Scale bar: 10 μm. (TIFF) [file ppat.1010434.s007.tiff]

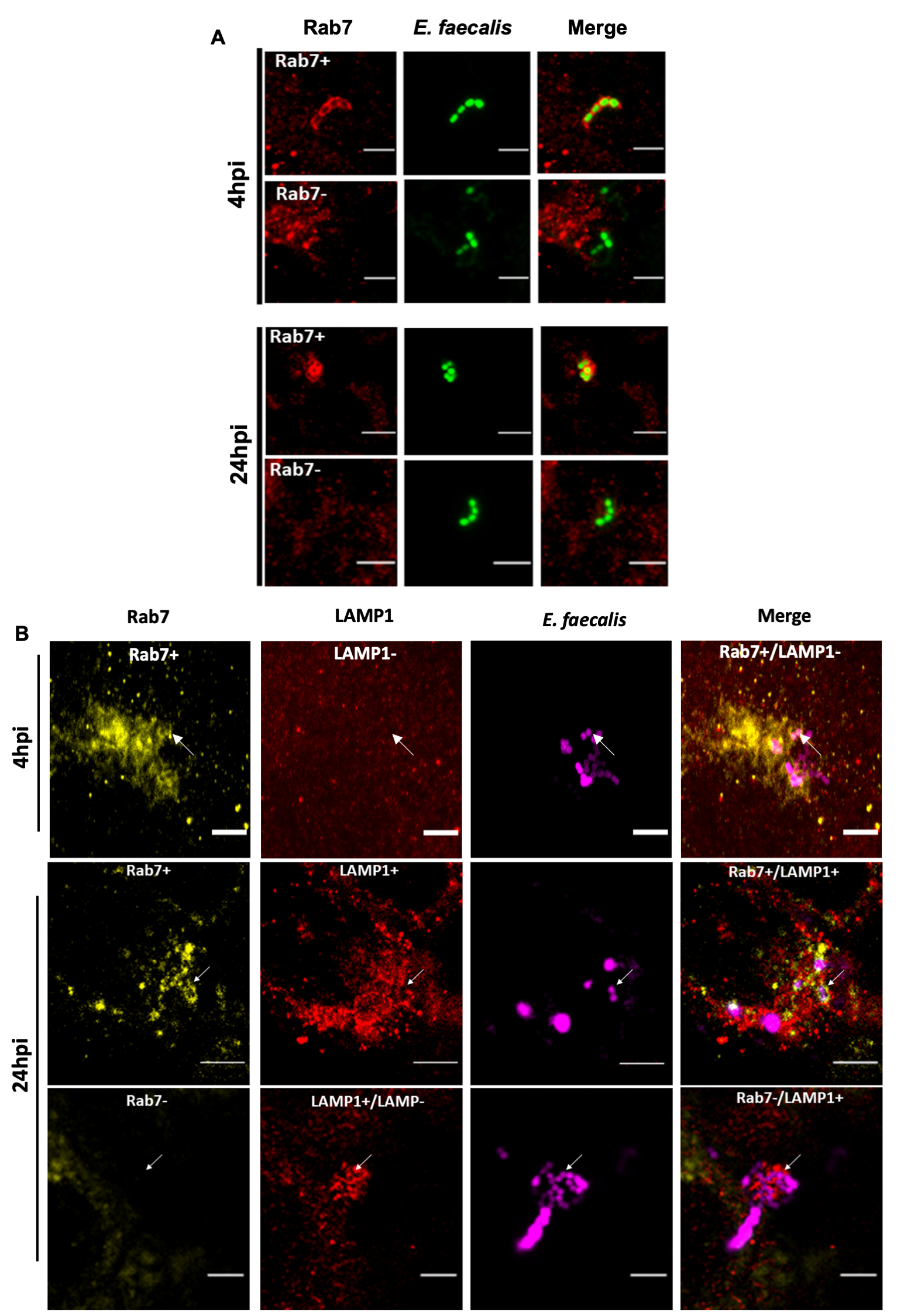

Supplement: S8 Fig — E. faecalis is found in heterogeneously labelled Rab7/LAMP1 compartments. (A) CLSM of infected HaCaTs with fluorescent labelling of Rab7 (late endosome) and fluorescent E. faecalis (pDasherGFP). Images show examples of Rab7+ and Rab7- compartments. Green, E. faecalis (pDasherGFP); and red, Rab7. Images shown are representative of 3 independent experiments. Scale bar: 5 μm. (B) CLSM of infected HaCaTs with fluorescent labelling of Rab7 and LAMP1 (late endosome) and fluorescent E. faecalis (pDasherGFP). Pink, E. faecalis (pDasherGFP); yellow, Rab7; red, LAMP1. Images show examples of Rab7+/LAMP1- at 4 hpi (top panel), Rab7+/LAMP1+ (middle panel), LAMP1+/Rab7- and LAMP1+/Rab7- (Bottom panel) compartments. Images shown are representative of 3 independent experiments. Scale bar: 5 μm. White arrows indicate areas of interest for E. faecalis-containing compartments. (TIFF) [file ppat.1010434.s008.tiff]

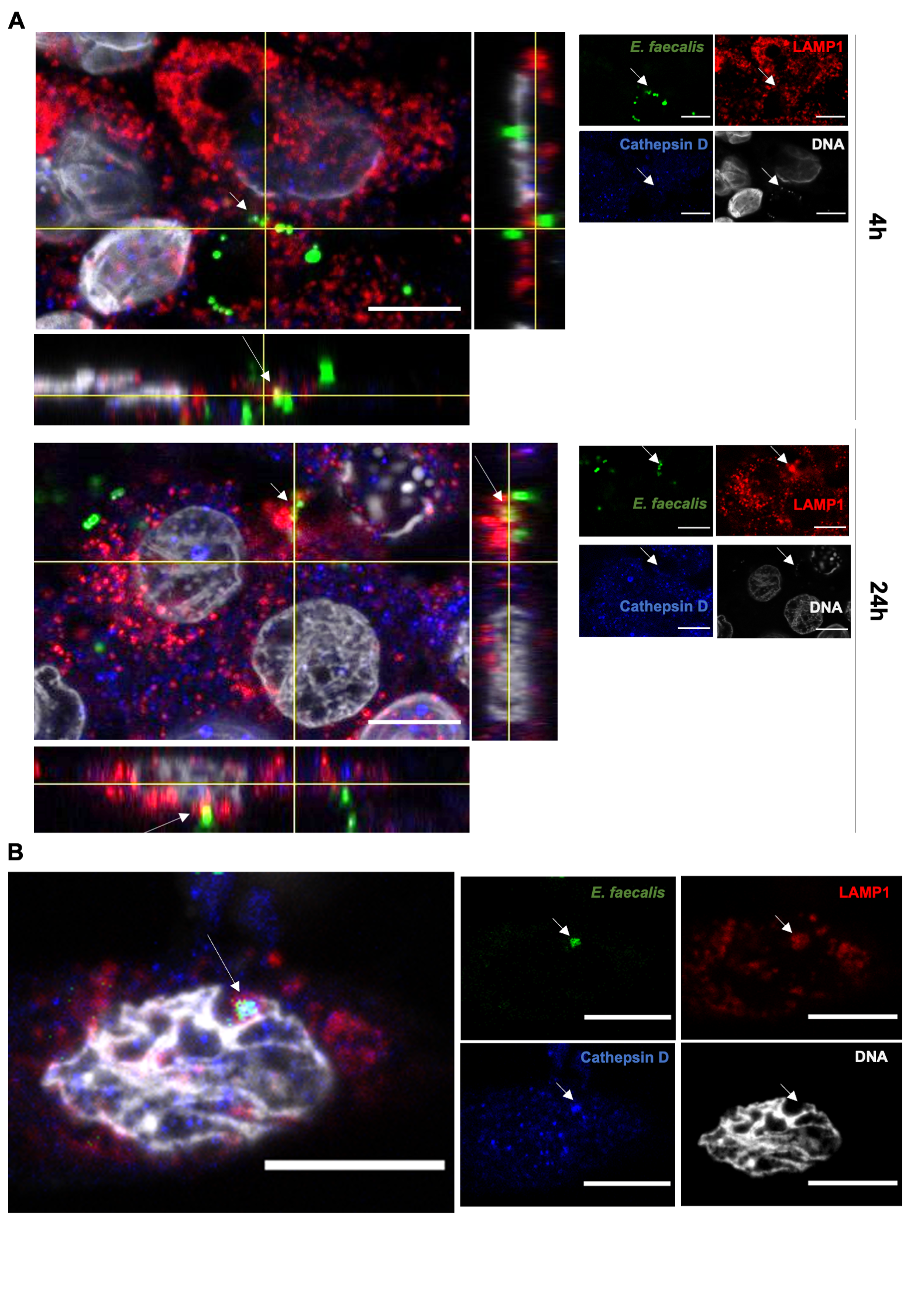

Supplement: S9 Fig — E. faecalis is rarely found in compartments that contain Cathepsin D. (A) CLSM Orthogonal views and individual channels of E. faecalis within keratinocytes labelled with antibodies against Cathepsin D and LAMP1 (monoclonal antibody) at 4 h and 24 hpi. Examples of E. faecalis colocalizing with LAMP1 but not with Cathepsin D can be observed (white arrows). White, dsDNA stained with Hoechst 33342; green, E. faecalis (pDasherGFP); red, LAMP1; and blue, Cathepsin D. Images are representative of 3 independent experiments. Scale bar: 10 μm. (B) Rare example of E. faecalis within keratinocyte colocalizing with Cathepsin D (white arrow). Keratinocytes were labelled with antibodies against Cathepsin D and LAMP1 (monoclonal antibody) at 24 hpi. White, dsDNA stained with Hoechst 33342; green, E. faecalis (pDasherGFP); red, LAMP1; blue, Cathepsin D. Images are representative of 3 independent experiments. Scale bar: 10 μm. (TIFF) [file ppat.1010434.s009.tiff]

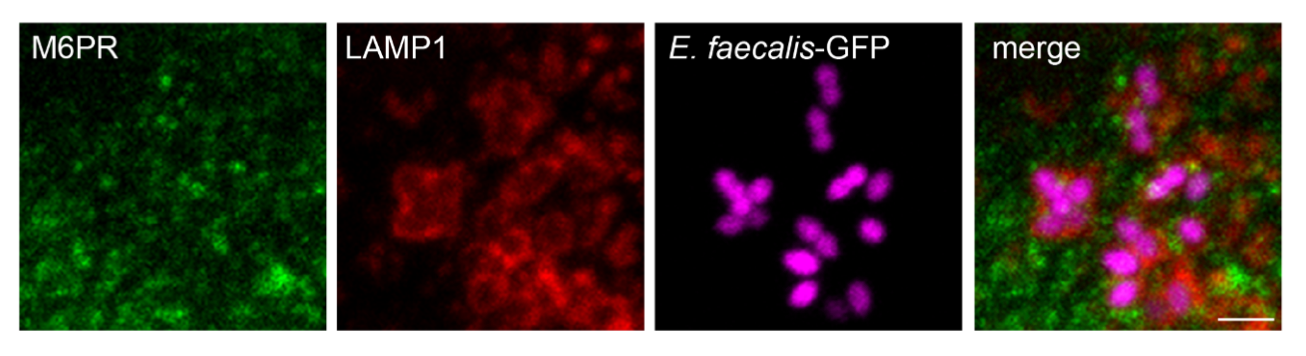

Supplement: S10 Fig — Internalized E. faecalis persist within late endosomal compartments. CLSM of infected HaCaTs stained with antibodies against M6PR (late endosome) and LAMP1 (late endosome/lysosome; polyclonal antibody) at 24 hpi. Images are maximum intensity projections of 4–5 optical sections (~2 μm z-volume) and are representative of 3 independent experiments. Scale bar: 2 μm. (TIFF) [file ppat.1010434.s010.tiff]

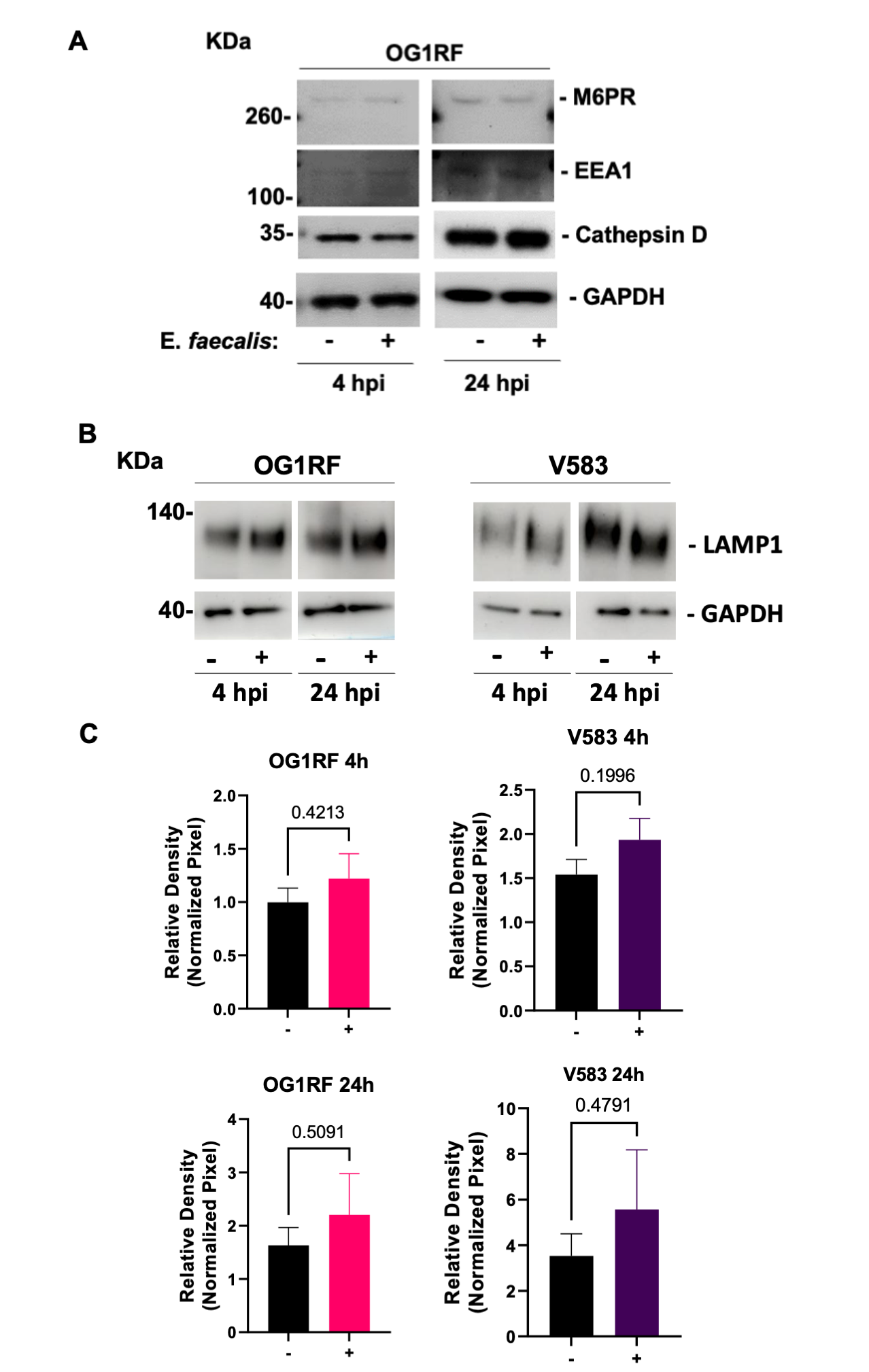

Supplement: S11 Fig — E. faecalis infection of keratinocytes does not alter expression of other endosomal proteins. (A) Whole cell lysates analyzed by immunoblot with antibodies α-M6PR, α-EEA1, α-CathepsinD, and α-GAPDH. HaCaT cells were incubated with (+) and without (-) E. faecalis OG1RF for 4 hpi and 24 hpi. Images shown are representative of 3 biological replicates. (B) Whole cell lysates analyzed by immunoblot with monoclonal antibody α-LAMP1 and α-GAPDH. HaCaT cells were incubated with (+) and without (-) E. faecalis OG1RF and V583 for 4 hpi and 24 hpi. Images shown are representative of 5 biological replicates. (C) Relative density of the bands of interest were normalized against loading control (GAPDH). Error bars represent biological replicates and mean ​± SEM from at least 3 independent experiments. Statistical analysis was performed using unpaired T-test with Welch’s correction. (TIFF) [file ppat.1010434.s011.tiff]

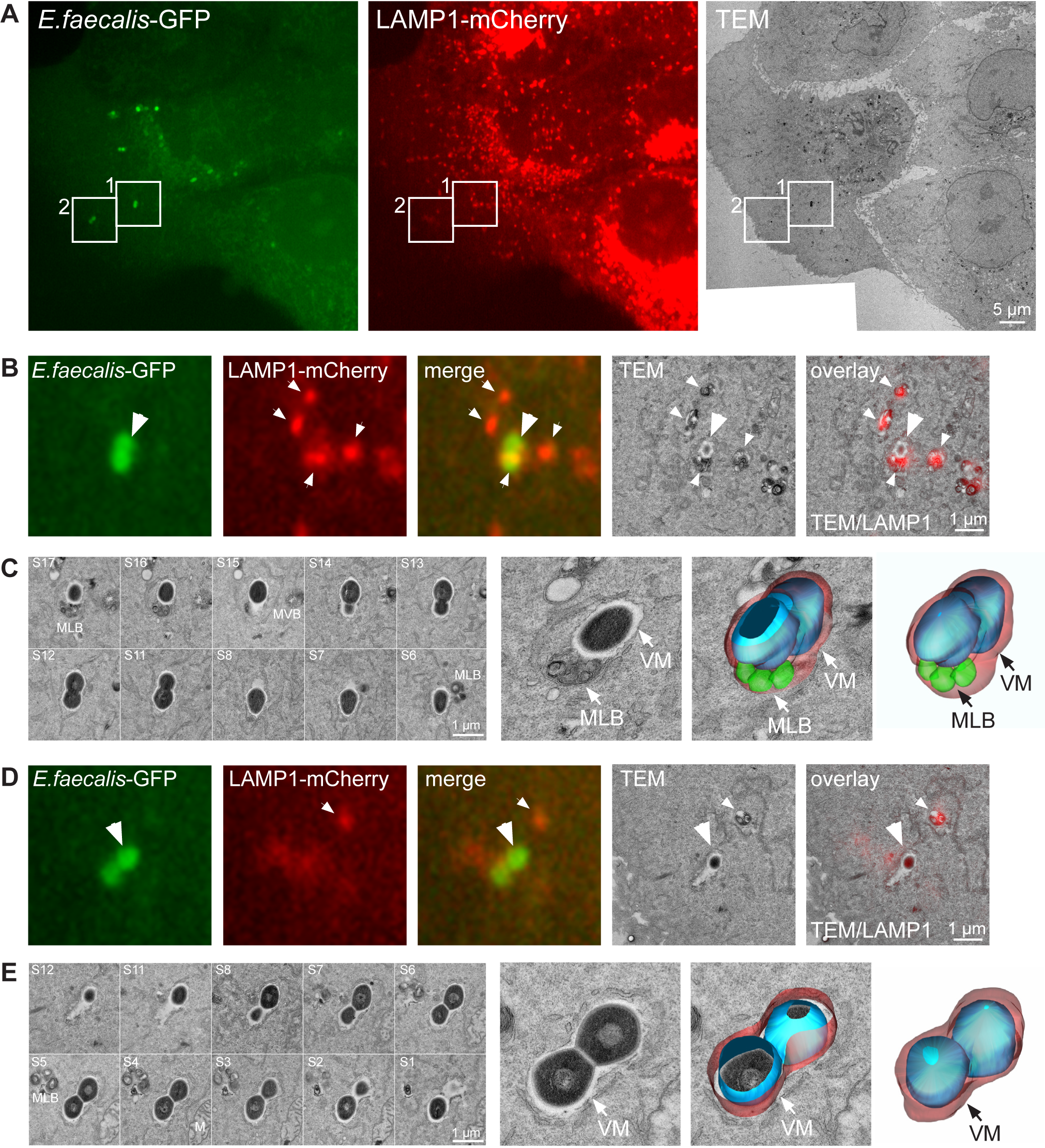

Supplement: S12 Fig — (A) Spinning disk confocal microscopy and correlative TEM of HaCaTs stably expressing LAMP1-mCherry infected with E. faecalis-GFP at 18 hpi. Confocal images are maximum intensity projections of 4–5 optical sections (~2 μm z-volume). (B) Enlarged views of area 1 highlighted in (A). (C) Serial section TEM and 3D surface rendering of the area shown in (B). (D) Enlarged views of area 2 highlighted in (A). (E) Serial section TEM and 3D surface rendering of the area shown in (D). Large arrowheads indicate E. faecalis containing vacuoles, small arrows indicate LAMP1+ compartments. VM: vacuolar membrane (VM); MLB: multilamellar body. An E. faecalis containing vacuole containing a LAMP1+ve MLB is shown in (B and C), while the E. faecalis containing vacuole shown in (D and E) is LAMP1-ve and does not contain an MLB (data pertinent to Fig 5F-H). (TIF) [file ppat.1010434.s012.tif]

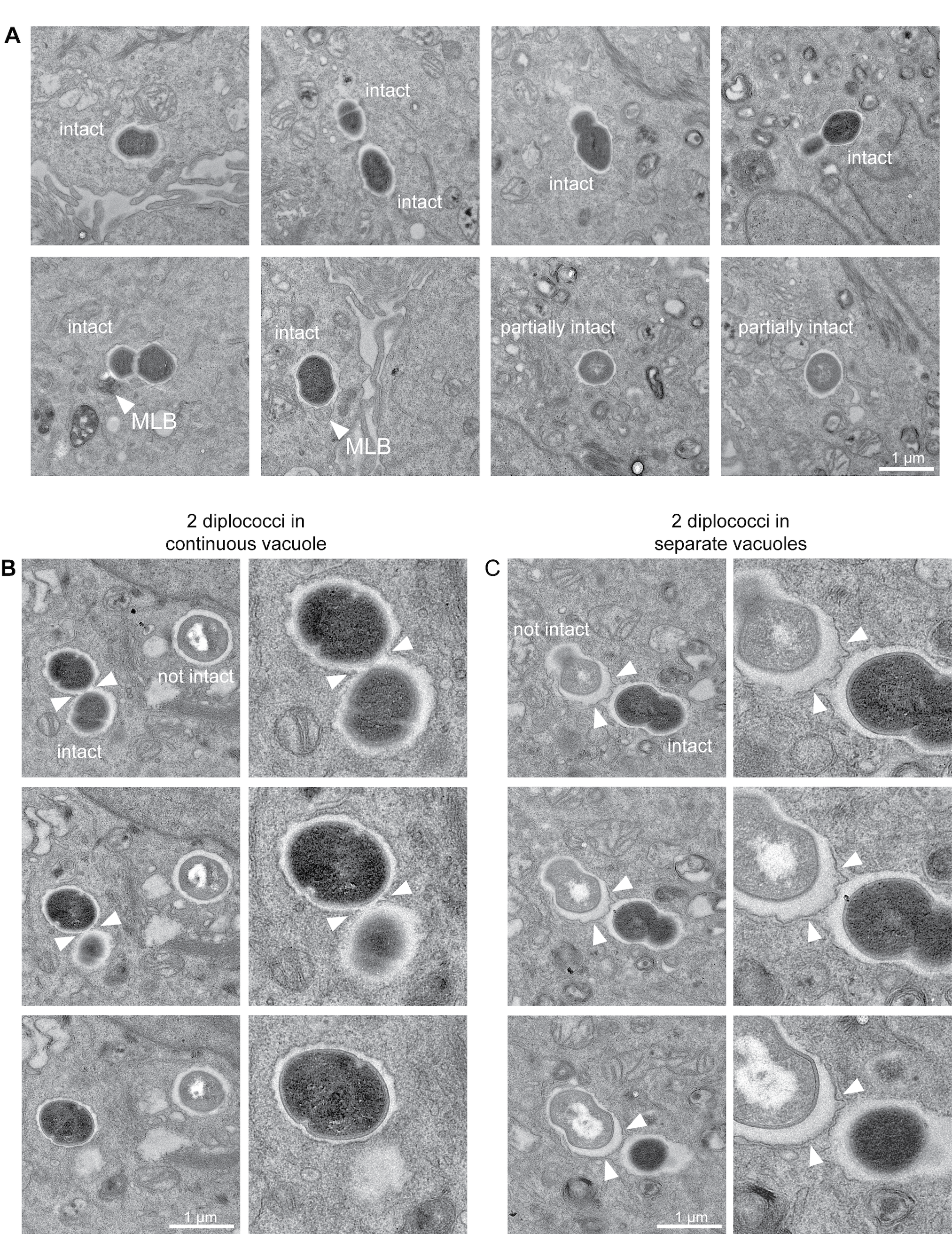

Supplement: S13 Fig — (A) Representative high magnification TEM images of E. faecalis containing vacuoles. Intact and partially intact bacteria are shown. Two examples of vacuoles containing MLBs are shown. (B and C) Serial section TEM analysis of E. faecalis containing vacuoles. (B) Two E. faecalis residing in a shared vacuole (area identical to that shown in Fig 7D). Note the continuity of the vacuolar lumen indicated by the two arrowheads. (C) Two E. faecalis residing in separate vacuoles. Note that the two vacuoles are separated by a vacuolar membrane, indicated by the two arrowheads. (TIF) [file ppat.1010434.s013.tif]
