# Supplementary material for: Enterococcus faecalis alters endo-lysosomal trafficking to replicate and persist within mammalian cells
Source: PLoS Pathog. 2022 Apr 7;18(4):e1010434. doi: 10.1371/journal.ppat.1010434 (PMC9017951; doi:10.1371/journal.ppat.1010434)
Supplement: S1 Table — HaCaT cells were incubated with various pharmacological inhibitors at the concentration used in antibiotic protection assays and subsequently assessed for viability using the AlamarBlue cell viability reagent. For cytochalasin D and latrunculin A, cells were incubated with the inhibitor for 24 h prior to assessment of viability. For wortmannin, colchicine, nystatin and dynasore, cells were incubated with the inhibitor for 4 h. Inhibitors resulting in HaCaT viability above 80% were considered as non-cytotoxic. (DOCX) [file ppat.1010434.s014.docx]

**S1 Table. Viability of HaCaT cells upon treatment with inhibitors**

HaCaT cells were incubated with various pharmacological inhibitors at the concentration used in antibiotic protection assays and subsequently assessed for viability using the AlamarBlue cell viability reagent. For cytochalasin D and latrunculin A, cells were incubated with the inhibitor for 24 h prior to assessment of viability. For wortmannin, colchicine, nystatin and dynasore, cells were incubated with the inhibitor for 4 h. Inhibitors resulting in HaCaT viability above 80% were considered as non-cytotoxic.

| **Inhibitor** | **Concentration** | **Viability** |
| --- | --- | --- |
| Cytochalasin D | 1 µg/ml | 88.5% |
| Latrunculin A | 0.25 µg/ml | 93.4% |
| Wortmannin | 0.1 µg/ml | 96.3% |
| Colchicine | 10 µg/ml | 80.0% |
| Nystatin | 25 μg/ml | 92.6% |
| Dynasore | 25 μg/ml | 76.2% |
