# Supplementary material for: Enterococcus faecalis alters endo-lysosomal trafficking to replicate and persist within mammalian cells
Source: PLoS Pathog. 2022 Apr 7;18(4):e1010434. doi: 10.1371/journal.ppat.1010434 (PMC9017951; doi:10.1371/journal.ppat.1010434)
Supplement: S2 Table — (DOCX) [file ppat.1010434.s015.docx]

**S2 Table. Primers used in this study.**

| **Purpose** | **Primer Type** | **Annealing Temperature (°C)** | **Primer Sequences (5’-3’)** |
| --- | --- | --- | --- |
| Lamp1 sub-cloning | F  R | 72°C  72°C | ATGGCGGCCCCCGGCA  GATAGTCTGGTAGCCTGCGTGACTCCTCT |
| Lamp1 linker addition | F  R | 72.7°C  70.5°C | GACAGCGCTACCATGGCGGCCCC  GCTCACCATGGTGGCGATAGTCTGGT |
| pEF1-α mCherry-N1 linearization | F  R | 63.9°C  61.5°C | GCCACCATGGTCAGCAAGGGC  GGTAGCGCTAGCGTCACGACA |
| Colony PCR verification | F  R | 60.8°C  61.5°C | TCCATTTCAGGTGTCGTGACGCT  CTACTTGTACAGCTCCATGCCG |
