# Supplementary material for: Enterococcus faecalis alters endo-lysosomal trafficking to replicate and persist within mammalian cells
Source: PLoS Pathog. 2022 Apr 7;18(4):e1010434. doi: 10.1371/journal.ppat.1010434 (PMC9017951; doi:10.1371/journal.ppat.1010434)
Supplement: Attachment — Submitted filename: 220221_ Response to Reviewers.pdf [file ppat.1010434.s017.pdf]

Editor points and summary response

1. The authors use the classical gentamicin exclusion assay to assess intracellular bacteria. As indicated by reviewer 1 and 3, no data is presented to show that extracellular bacteria are killed in your specific model system. Such verification needs to be added to the manuscript to enable proper interpretation and conclusions of the results.

We have now included antibiotic killing data for the *E. faecalis* strains used in this study in **Supplementary Figure 1**.

1) **Panels A and F (supernatant).** We always sample the assay supernatant following infection and 21 h of antibiotic treatment (just prior to washing and lysis) and observe *zero* OG1RF CFU, and zero or nearly zero V583 CFU. This had been described with "data not shown" in the previous submission. Representative data are shown in these panels, confirming that extracellular bacteria are killed in our specific model system.
2) **Panels B and G.** Planktonic bacterial culture (in the absence of mammalian cells) treated with antibiotic concentrations used in the gentamicin protection assay (500ug/ml each of gentamicin and penicillin) resulted in killing of 99.9% of bacteria by 1 h and 21 h antibiotic treatment for OG1RF and V583, respectively.
3) **Panels A and F (PBS wash).** In response to reviewer #3, a suggestion which we appreciated and hadn't considered previously, we also found very few *E. faecalis* CFU in the final (3rd) PBS wash post antibiotic treatment (<0.1% compared to the intracellular population). While this number is not zero for most of the samples at the standard MOI 100 used throughout this manuscript, we suspect is derived from bacteria-containing host cells sloughing off during the wash.

In summary, from these data we conclude that our gentamicin exclusion assay is well validated and that the minimal number of extracellular CFU that can be recovered under the conditions described above does not significantly affect the final interpretation of the results.

2. As indicated by all three reviewers, the number of replicates used for analyses are generally low and due to their spread in several assays this makes interpretation difficult and some of the conclusion premature.

All experiments have a minimum of three independent biological replicates. All CLSM visualization score analyses include a minimum of 8 images analyzed per data set, with 2-3 images per biological replicate. Western blots were repeated at least three independent times.

3. There is insufficient evidence to conclude that Enterococci replicate inside cells. First, the criteria used during imaging to determine whether bacteria are intracellular or not are clear. This combined with the reported decrease in intracellular bacteria over time, a decrease of bacteria in LAMP-positive vacuoles over times, and lack of evidence for replication in the CLEM analyses do not support the conclusion that bacteria replicate intracellularly. All three reviewers suggest approaches to address this issue.

We now provide <u>new data</u> supporting intracellular replication *in vivo* and *in vitro.* Our new data also adds a second cell line (RAW 264.7 macrophages) to the previous analysis which

only examined intracellular replication in HaCaT keratinocytes. **Figure 3C-D** contains these new panels in which we show BrdU (incorporated into newly replicated DNA) and RADA (incorporated into newly replicated bacterial cell wall) labelling data of *E. faecalis* inside HaCaT and RAW264.7 cells, and BrdU labelling of intracellular bacteria within infected murine wound tissue.

We chose to include data using these *two* approaches to demonstrated intracellular replication because BrdU labelling and staining, while a widely used tool to assess active cellular replication, is technically challenging and has its limitations. For example, the required acid treatment to allow for DNA exposure and anti-BrdU antibody binding is harsh and can influence the outcome of the technique affecting bacterial cell shape and different labelling alignments when acquiring the image, particularly in our *in vivo* samples. We are confident that the fluorescent GFP-expressing bacterial clusters we observe are, in fact, bacteria, because the mammalian cells have low auto-fluorescence in the green channel. Similarly, controls for non-incorporation of BrdU in non-replicating bacteria also confirm limitations of the technique, in that we do not achieve 100% labelling of replicating bacteria as we do for RADA (new **Supplementary Figure 5**); nonetheless, controls show that BrdU labelling (while inefficient) is only incorporated by replicating bacteria. Taken together, the data support that *E. faecalis* intracellular replication occurs within mammalian cells.

4. In the experiments addressing bacterial entry mechanisms, information showing that the inhibitors used are not toxic to cells or bacteria (except for Dynasore) and that they function as specified in your specific cell system would be needed to draw clear conclusions.

We appreciate this comment, and we provide cytotoxicity data ("data not shown" in the previous submission) for the used compounds in the new **Table S1**. In short, only one compound (Dynasore) results in >20% cytotoxicity, so we very conservatively interpret results of Dynasore experiments. For even low-level cytotoxicity to confound the analysis (such that cytotoxicity would release intracellular bacteria in the antibiotic-containing medium where they would be killed), one would expect greater toxicity to correlate with a reduction in intracellular CFU, but that is not what we see. These compounds are well documented in the literature for this type of test with and without bacteria as shown in the references below, which was used to guide our choice of drug concentrations in these experiments:

Cytochalasin D (1.97 μM)

Disruption of the actin cytoskeleton has been observed with pre-treatment of HaCaT cells using 2 μM cytochalasin D within 30 minutes treatment. It is also reported that there is no cytotoxic effects with 2 μM cytochalasin D treatment on HaCaT cells. This information is supported by the following publications:

Blase C, Becker D, Kappel S, Bereiter-Hahn J. Microfilament dynamics during HaCaT cell volume regulation. Eur J Cell Biol. 2009 Mar;88(3):131-9. doi: 10.1016/j.ejcb.2008.10.003. Epub 2008 Nov 25. PMID: 19036471.

Kang J, Chen W, Xia J, Li Y, Yang B, Chen B, Sun W, Song X, Xiang W, Wang X, Wang F, Bi Z, Wan Y. Extracellular matrix secreted by senescent fibroblasts induced by UVB promotes cell proliferation in HaCaT cells through PI3K/AKT and ERK signaling pathways. Int J Mol Med. 2008 Jun;21(6):777-84. PMID: 18506372.

Latrunculin A (0.25 µg/mL or 0.593 µM)

Coué M, Brenner SL, Spector I, Korn ED. Inhibition of actin polymerization by latrunculin A. FEBS Lett. 1987 Mar 23;213(2):316-8. doi: 10.1016/0014-5793(87)81513-2. PMID: 3556584.

> *(…) the reaction in vitro is about 0.2 µM whereas the effects of the drug on cultured cells are detectable at concentrations in the medium of 0.1–1 µM.*

Jeong SY, Martchenko M, Cohen SN. Calpain-dependent cytoskeletal rearrangement exploited for anthrax toxin endocytosis. Proc Natl Acad Sci U S A. 2013 Oct 15;110(42):E4007-15. doi: 10.1073/pnas.1316852110. Epub 2013 Oct 1. PMID: 24085852; PMCID: PMC3801034.

> *RAW264.7 cells were preincubated with or without 1 µM of LatA for 40 min at 37 °C.*

Affentranger S, Martinelli S, Hahn J, Rossy J, Niggli V. Dynamic reorganization of flotillins in chemokine-stimulated human T-lymphocytes. BMC Cell Biol. 2011 Jun 22;12:28. doi: 10.1186/1471-2121-12-28. PMID: 21696602; PMCID: PMC3131241.

> *T-lymphoblasts were incubated for 45 minutes at 37°C (control) or were preincubated for 30 minutes without or with latrunculin A (lat; 1 µM)*

Boettcher JP, Kirchner M, Churin Y, Kaushansky A, Pompaiah M, Thorn H, Brinkmann V, Macbeath G, Meyer TF. Tyrosine-phosphorylated caveolin-1 blocks bacterial uptake by inducing Vav2-RhoA-mediated cytoskeletal rearrangements. PLoS Biol. 2010 Aug 24;8(8):e1000457. doi: 10.1371/journal.pbio.1000457. PMID: 20808760; PMCID: PMC2927421.

> *Human cervix carcinoma cell line ME-180… Cells were treated with … 1 µM Cyt D (Sigma Aldrich, St. Louis, MO, USA) or 100 nM Lat A (Biomol, Hamburg, Germany) for 30 min.*

Wortmannin (0.1 µg/mL = 0.233 µM)

Edwards AM, Potter U, Meenan NA, Potts JR, Massey RC. Staphylococcus aureus keratinocyte invasion is dependent upon multiple high-affinity fibronectin-binding repeats within FnBPA. PLoS One. 2011 Apr 22;6(4):e18899. doi: 10.1371/journal.pone.0018899. PMID: 21526122; PMCID: PMC3081306.

Siemens N, Patenge N, Otto J, Fiedler T, Kreikemeyer B. Streptococcus pyogenes M49 plasminogen/plasmin binding facilitates keratinocyte invasion via integrin-integrin-linked kinase (ILK) pathways and protects from macrophage killing. J Biol Chem. 2011 Jun 17;286(24):21612-22. doi: 10.1074/jbc.M110.202671. Epub 2011 Apr 26. PMID: 21521694; PMCID: PMC3122219.

Nam HJ, Park YY, Yoon G, Cho H, Lee JH. Co-treatment with hepatocyte growth factor and TGF-beta1 enhances migration of HaCaT cells through NADPH oxidase-dependent ROS generation. Exp Mol Med. 2010 Apr 30;42(4):270-9. doi: 10.3858/emm.2010.42.4.026. PMID: 20177149; PMCID: PMC2859326.

Kang J, Chen W, Xia J, Li Y, Yang B, Chen B, Sun W, Song X, Xiang W, Wang X, Wang F, Bi Z, Wan Y. Extracellular matrix secreted by senescent fibroblasts induced by UVB promotes cell proliferation in HaCaT cells through PI3K/AKT and ERK signaling pathways. Int J Mol Med. 2008 Jun;21(6):777-84. PMID: 18506372.

Colchicine (10 µg/mL = 25 µM)

Edwards AM, Potter U, Meenan NA, Potts JR, Massey RC. Staphylococcus aureus keratinocyte invasion is dependent upon multiple high-affinity fibronectin-binding repeats within FnBPA. PLoS One. 2011 Apr 22;6(4):e18899. doi: 10.1371/journal.pone.0018899. PMID: 21526122; PMCID: PMC3081306.

Ellington JK, Reilly SS, Ramp WK, Smeltzer MS, Kellam JF, Hudson MC. Mechanisms of Staphylococcus aureus invasion of cultured osteoblasts. Microb Pathog. 1999 Jun;26(6):317-23. doi: 10.1006/mpat.1999.0272. PMID: 10343060.

Kuhn M. The microtubule depolymerizing drugs nocodazole and colchicine inhibit the uptake of Listeria monocytogenes by P388D1 macrophages. FEMS Microbiol Lett. 1998 Mar 1;160(1):87-90. doi: 10.1111/j.1574-6968.1998.tb12895.x. PMID: 9495017.

Dynasore (25 µg/mL = 77.6 µM)

Dynasore has been previously used in HaCaT cells, and it has been shown that dynamin-dependent transferrin uptake is blocked in HaCaT cells which were pre-treated with 40 µM dynasore. 80 µM dynasore was also used on HaCaT cells and stated in-text that there were no cytotoxic effects. This information is supported by the following publications:

Rahn E, Petermann P, Hsu MJ, Rixon FJ, Knebel-Mörsdorf D. Entry pathways of herpes simplex virus type 1 into human keratinocytes are dynamin- and cholesterol-dependent. PLoS One. 2011;6(10):e25464. doi: 10.1371/journal.pone.0025464. Epub 2011 Oct 12. PMID: 22022400; PMCID: PMC3192061.

Völlner F, Ali J, Kurrle N, Exner Y, Eming R, Hertl M, Banning A, Tikkanen R. Loss of flotillin expression results in weakened desmosomal adhesion and Pemphigus vulgaris-like localisation of desmoglein-3 in human keratinocytes. Sci Rep. 2016 Jun 27;6:28820. doi: 10.1038/srep28820. PMID: 27346727; PMCID: PMC4922016.

Nystatin (25 µg/mL = 27 µM)

There are limited studies showing the effect of nystatin on HaCaT cells. Depletion of cholesterol by nystatin has been used to inhibit TGF-β-induced epithelial-mesenchymal transition in HaCaT cells, demonstrating that HaCaT cells are sensitive to the cholesterol-depleting effects of nystatin. Note that the concentrations or time-points used in these experiments (25 µg/mL for 36 hours, 50 µg/mL for 1 hour) are different from those used in our experiments (25 µg/mL for 30 minutes). This information is supported by the following publications:

Rahn E, Petermann P, Hsu MJ, Rixon FJ, Knebel-Mörsdorf D. Entry pathways of herpes simplex virus type 1 into human keratinocytes are dynamin- and cholesterol-dependent. PLoS One. 2011;6(10):e25464. doi: 10.1371/journal.pone.0025464. Epub 2011 Oct 12. PMID: 22022400; PMCID: PMC3192061.

Chen Y, Wang S, Lu X, Zhang H, Fu Y, Luo Y. Cholesterol sequestration by nystatin enhances the uptake and activity of endostatin in endothelium via regulating distinct endocytic pathways. Blood. 2011 Jun 9;117(23):6392-403. doi: 10.1182/blood-2010-12-322867. Epub 2011 Apr 11. PMID: 21482707.

Calay D, Vind-Kezunovic D, Frankart A, Lambert S, Poumay Y, Gniadecki R. Inhibition of Akt signaling by exclusion from lipid rafts in normal and transformed epidermal keratinocytes. J Invest Dermatol. 2010 Apr;130(4):1136-45. doi: 10.1038/jid.2009.415. Epub 2010 Jan 7. PMID: 20054340.

5. Finally, all reviewers request additional information to clarify the results on intracellular trafficking and manipulation of Rab5 and Rab7. Information about how co-localization was determined is missing and how the authors have excluded the possibility that potential transient markers, such as Rab5 and Rab7 were never associated with the bacterial vesicles are not stated. This will be important to specify.

Co-localization was determined by visual inspection using orthogonal views and fluorescence intensity profile and 3D image projections generated on Imaris 9.0.2. We now include new data (**Figure 4A-D and 5B-C**) demonstrating and quantifying colocalization of labelled proteins of interest with fluorescent *E. faecalis* with bacteria over a linear segment (histograms). An example of a 3D projection can be visualized in new **Supplementary Video 1** of BrdU labelling of ex-vivo cell images. We also added additional examples of orthogonal views for relevant figures (**Supplementary Figures 7 and 9**).

We did not previously discuss the possibility that intracellular *E. faecalis*-containing compartments fail to associate with transient markers such as Rab5 and Rab7, in part because we did not feel confident making such an inference based on the datasets we had at the time (with limited timepoints only at 4 and 24 hpi). To address this more thoroughly, we investigated Rab5 and Rab7 labelling at time points starting at 30min, 1h and 3h (**Figure 4A-D**). The dynamics of these two small GTPases is quite useful since Rab5 is exchanged with Rab7 via Rab conversion. In instances where Rab5 does not associate with *E. faecalis*-containing compartments, we expected to see Rab7 close or associated to the compartment, which is why we also analyzed the compartments for Rab5/Rab7 labelling (together or in close proximity). This approach revealed that *E. faecalis* displays heterotypic intracellular trafficking. At every timepoint examined, we observe some *E. faecalis* associated with markers of early endosomes (eg. Rab5) and some *E. faecalis* associated with markers of late endosomes (eg. Rab5/7 or Rab7 alone), indicating normal trafficking. At the same time, at each timepoint we also observe another subset of *E. faecalis* showing no association with Rab5 or Rab7 (**Figures 4A-D and Supplementary Figure 7**). From these new data, we can now suggest with more confidence that a subset of intracellular *E. faecalis* may never associate with canonical endo-lysosomal compartments."/

R1 - Summary

This paper from Tay et al. explores the interesting issue of how Enterococcus faecalis persists chronically in wounds, which is important knowledge that could be leveraged for developing novel treatments in what is a very important clinical indication from a healthcare burden perspective. As the authors themselves recognize, the main results are not completely new – a number of other publications have shown that E. faecalis invades and persists in different kinds of cells (both immune and non-immune). The diversity of strategies that might be used by E. faecalis to persist inside keratinocytes is the novelty, but clarification and a number of confirmatory experiments are required to support these findings. As the results stand, the conclusions are not supported. The risk is that if those

experiments are improved, it might be the case that the main (novel) conclusions are not supported.

We appreciate the overall perspective given here about our paper. We have now added new information that we believe re-enforces our initial observations and conclusions, and the novelty of the work both in that *E. faecalis* replicates intracellularly and subverts canonical endo-lysosomal trafficking and maturation in order to persist within this niche.

R1 - Major

Most of the experiments needed are presented in the paper, so it's not a case that major experiments are needed, but the issue is that a lot of them are not rigorous enough and would have to be repeated/controls included/more replicates etc. If those experiments are improved, it might be the case that the main (novel) conclusions are not supported.

In the previous submission, all experiments were performed in a minimum of three independent biological replicates. We have taken care in this revision so that is clear.

Our paper has been improved with an increase in the data provided. Data on the heterotypic intracellular trafficking of *E. faecalis* provided before (e.g. Rab7 and LAMP1) have been corroborated and expanded with new data on Rab5, Rab7, LAMP1 and Cathepsin D. Moreover, we have added new data demonstrating potential intracellular replication of *E. faecalis* in murine wound tissue.

1. There is insufficient evidence to support the idea that E. faecalis replicates inside cells. CFUs go down over time; many experiments do not have parallel confocal stacks to confirm intracellularity during the experiment; that bacteria may merely be re-entering anew (rather than persisting) has not been ruled out; some experiments suffer from a lack of information about MOI, antibiotic concentration and kill curves, and bacteria growth curves; there are insufficient controls to cover the well-known pitfalls of the protection assay (including incomplete bacterial killing; cell permeability at high concentrations; and protection due to extracellular biofilms). The details for the above observations are provided in Part III.

Addressing each of these concerns point by point:
<u>1a. Insufficient evidence to support the idea that *E. faecalis* replicates inside cells.</u>
Please see above, response to editor point 3, for a summary of our newly included evidence in support of intracellular replication.

<u>1b. CFUs go down over time.</u>
Intracellular replication within a subset of infected cells is not inconsistent with a slow decrease in intracellular CFU in the entire population of mammalian cells. Particularly in the *in vitro* model, in which infected cells supporting intracellular replication will lyse at some point, we expect that the released bacteria will be killed by antibiotics in the media rather than reinfect neighbouring cells. Also, slow decrease in CFU over time could be a net effect of intracellular killing vs intracellular replication -- killing may be happening at a faster rate than the bacteria can replicate. One could imagine that without replication, the intracellular bacteria population would have decreased more rapidly and the extended intracellular

persistence reported herein may not have been observed. This hypothesis was in fact proposed by Gentry-Weeks et al (1999) when they showed, for the first time, evidence of extended *E. faecalis* persistence in murine peritoneal macrophages. We do not yet know why we see a slow decline of intracellular bacteria in the *in vivo* model, and this is something we are working hard to understand – how does intracellular replication/persistence *in vivo* contribute to pathogenesis. This, we feel, is beyond the scope of this manuscript.

<u>1c. Many experiments do not have parallel confocal stacks to confirm intracellularity during the experiment.</u>
Additional orthogonal views showing intracellular bacteria were added to the paper as **Supplementary Figures 7 and 9**).

<u>1d.</u> <u>Bacteria may merely be re-entering anew (rather than persisting) has not been ruled out.</u>
Bacteria re-entering and reinfecting cells is expected *in vivo,* but not in our *in vitro* assays in which we maintained the antibiotic pressure throughout as explained in 1b above.

<u>1e. Some experiments suffer from a lack of information about MOI, antibiotic concentration and kill curves, and bacteria growth curve.</u>

Unless otherwise stated, we used MOI 100 in all our *in vitro* experiments. This MOI is not toxic for the HaCaT cells within the infection times used (**Supplementary Figure 2**). Antibiotic killing in the assays and kill curves are now included in **Supplementary Figure 1** and summarized above in response to editor point 1. While both *E. faecalis* strains used in this study grow similarly in the cell culture media used, growth per se is not relevant because *all* of the experiments in this manuscript are done in the presence of antibiotics so no “growth” is happening.

<u>1f.</u> <u>There are insufficient controls to cover the well-known pitfalls of the protection assay (including incomplete bacterial killing; cell permeability at high concentrations; and protection due to extracellular biofilms.</u>

- Efficient bacterial killing by gentamicin and penicillin used in all of our antibiotic protection assays are now included in **Supplementary Figure 1** and summarized above in response to editor point 1.
- We cannot exclude the possibility that the mammalian cells become permeable to antibiotics, especially at the high concentrations that we require for *E. faecalis* killing. But even if they did become permeable, and this led to mammalian cell lysis and/or killing of intracellular bacteria, we’d expect to recover even fewer intracellular bacteria than we already do, which would not confound the conclusions of our study.
- The question about antibiotic protection due to extracellular biofilms is a good one. Indeed, in other work in the lab, we regularly use increased antibiotic tolerance as a key indication that biofilms are forming. However, our imaging data of intracellular *E. faecalis* (often within intracellular mammalian cell compartments) coupled with the antibiotic killing controls described above, strongly support the conclusion that the intracellular CFU that we enumerate are indeed intracellular and not simply antibiotic tolerant biofilms sitting on top of the host cells. Moreover, we tested a biofilm deficient mutant (in which the *ebp* pilus locus is disrupted) and did not see a change in intracellular CFU, indicating that in the context of our *in vitro* experiments, biofilm likely plays a minor or non-existent role, at least for the time points tested.

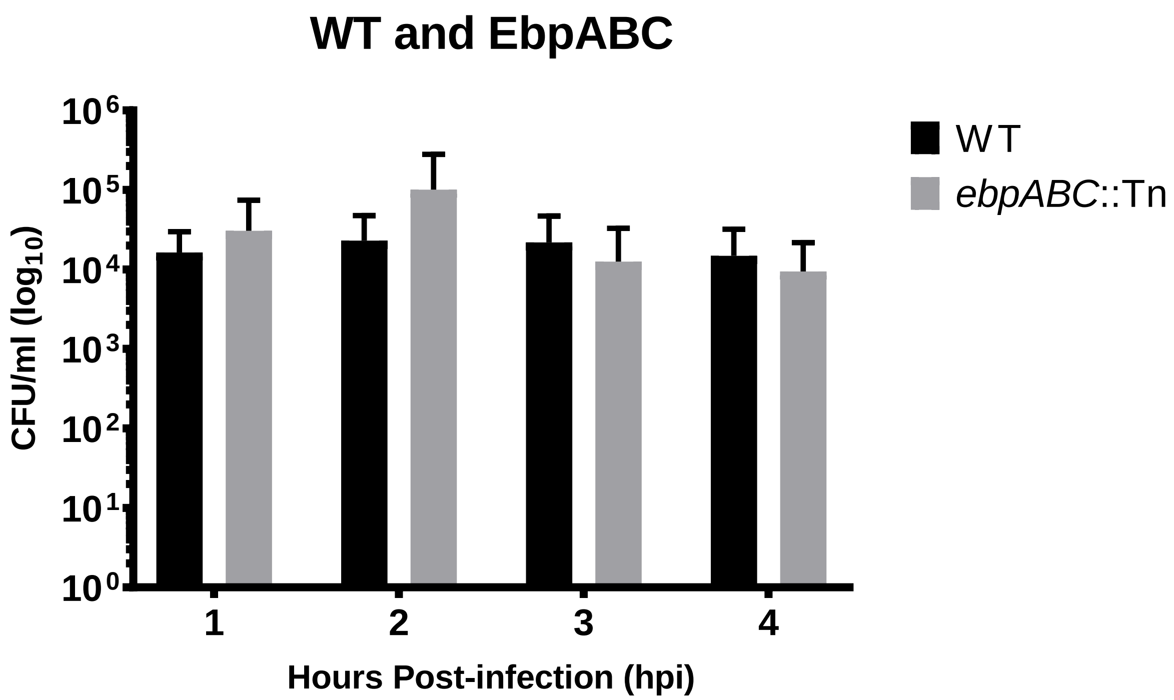

Figure 1. Recovered CFU numbers of *E. faecalis* OG1RF and *ebpABC*::Tn mutant from infected HaCaT cells.

2. The inhibitor and co-localization data are not convincing - The details for the above observations are provided in Part III.

Please see above, response to editor point 4.

3. A few experiments do not have enough biological replicates to give confidence that the findings are robust - The details for the above observations are provided in Part III.

All experiments had a minimum of three biological replicates.

R1 - Minor

- Statements saying that they are raising the issue that intracellular persistence may influence chronic E. faecalis infection should be removed or modified, since other studies had already raised that hypothesis. Therefore these authors are supporting those works but not trail-blazing the hypothesis.

We have amended the language and limit the speculation to chronic *E. faecalis* wound infections, which we do not believe has been raised in other studies, or to a role of intracellular replication in *E. faecalis* infections which has not been previously demonstrated.

- The absence of numbered lines in the document was very frustrating and hindered the review process.

Changed.

Title:

Needs to be altered or qualified, otherwise it is misleading, because:

- The authors don't have enough evidence to show that E. faecalis replicate in vivo (see more detailed comments below)

Please see above, response to editor point 3.

- Moreover, they only show one (very limited) experiment "during wound infection" in mice. If they wanted to focus the paper on wound infection, additional experiments are necessary to confirm the results (e.g. with epithelium organoids, or more mice experiments, or mimicking wounds in vitro in cell layers). In opposition, the majority of the results were obtained with cells/monolayers of keratinocytes without mimicking a wound.

This has been addressed with a title change to: *Enterococcus faecalis* alters endo-lysosomal trafficking to replicate and persist within mammalian cells.

Introduction:

This section is extremely short and superficial, focusing mainly on the discussion of a previous paper published by the authors and a summary of the results obtained in this work. It would have been good to see more details of other papers showing Enterococcus invasion to set the scene. Also, it is always good to give the clinical picture – why should we care about wound infection? What is the global burden both economically and from an incidence point of view?

We have now added a paragraph on intracellular trafficking of bacteria since the paper focuses on this scientific question. The wound context is not the major focus of the work and we think we have a good balance of wound background given its weight and the context of this study.

Minor comment: Enterococci should not be in italics.

Changed.

Results:

Intracellular E. faecalis are present within CD45+ and CD45- cells during mouse wound infection

- The antibiotic protection assay is not without its flaws. First, the antibiotic concentration used should be mentioned (at least as Supplementary material) and a dose response curve of antibiotic(s) used should be shown to assess if the concentration used was enough to kill all the bacteria. On the other hand, previous work by others has shown that very high concentrations of so-called impermeant antibiotics (even the gold standard gentamicin) can actually enter some cell types – have the authors confirmed that this does not happen under their conditions? Does the antibiotic have any cytotoxic effect on the suspended cells? We need to know this to assess the results obtained regarding the intracellular bacteria quantification. Finally, and perhaps most critically, Enterococci can form biofilms, even in a

relatively short period of time, and biofilms would be largely resistant to antibiotics. Extracellular biofilms therefore could contribute to CFU post-treatment which could artificially inflate the estimates of intracellular bacteria.

Please see above, response to editor point 1, as well as 1e and 1f in the response to this reviewer's major comment 1.

- The area/depth of the wound harvested and the mean number of cells recovered should also be mentioned (in the Material & Methods and/or main text), to help the reader understand possible variations in the CFU counting.

The area of the harvested wound is approximately 1cm x 1cm as stated in the M&M. The mean number of cells sorted was of 10k, although this doesn't reflect the absolute number of cells within a dissociated wound. We have not counted the total number of cells in the excised wound tissue, and are not sure how this information would alter the interpretation of our data.

- A comment/discussion or possible explanation observed for the 2 different "subpopulations" of infected CD45- cells should be provided. This becomes important in light of the subsequent results obtained in vitro by the authors.

We suspect the dynamic cycle of "infection, persistence, replication, cell lysis and re-infection" could explain variations in the observed outcomes. The reviewer may be interested to know whether the subpopulations were distributed evenly across 3 independent experiments, so the disparities observed are not an artifact of an individual experiment. We also considered the hypothesis that different early immune responses in a subset of animals might shape the nature of persistent intra- versus extra-cellular persistence as reported by Hannan et al for UPEC in chronic cystitis (PLoS Pathogens 2010, Early Severe Inflammatory Responses to Uropathogenic *E. coli* Predispose to Chronic and Recurrent Urinary Tract Infection); however, we observed no evidence of heterogeneity in early immune response in the *E. faecalis* infected wound (Chong et al, JID 2017, *Enterococcus faecalis* modulates immune activation and slows healing during wound infection).

E. faecalis adheres to and enters keratinocytes

- As above, a dose response curve of antibiotic(s) used should be shown to assess if the concentration and treatment periods used (which varied from 1h to 21h depending on the experiment) were enough to kill all the bacteria

Please see above, response to editor point 1.

- The sentence "Parallel cytotoxicity …. (data not shown)" is vague and could be confusing. It should be better explained, since the subsequent experiments in Supp Fig. 1 and Fig. 2 were performed at 1, 2, 3 and 4 hpi.

Done.

- The authors' use of the strain V583 should be better contextualized and explained. It only appeared in this section (and Fig. 5) and the authors commented that it showed "even higher numbers within HaCat cells", although the bacteria recovered after 48h and 72h dropped dramatically (which is not explained). In addition, since it seems a better "persister", why was not used for all the other studies?

OG1RF is the most commonly used lab strain for *E. faecalis*, because it is genetically tractable. V583 is the most common VRE *E. faecalis* strain used in the lab and is sufficiently genetically different from OG1RF that is often used to assess strain specificity of a phenomenon, as we do here. We don't propose (and therefore don't comment) that one is more relevant than the other for these studies or this infection model. Many of our initial experiments were performed with OG1RF, and we only later came to appreciate that V583 might do even better in the intracellular niche. It was not practical (nor necessary, for the conclusions of this study) to repeat everything with V583. But we will certainly use v583 in follow-up studies.

- The MOIs used for the images presented in Fig 2 should be mentioned (main text and caption).

Done.

- A panel of images comparing the different MOIs and time points showed in Fig 2A and 2B should be presented, as well as showing in Z-stack what the authors consider to be "internalized" and "adhered" (e.g. with arrows). Moreover, given there's some point-spread going on, not all of the bacterial in the ortho views seem, in my opinion, to look particularly internalized at this resolution. As a minor point, it's quite hard to see the cross-hairs on some images. Maybe make them white?

Please see **Supplementary Figures 7 and 9** for images from multiple time points and with additional orthogonal views. Cross-hairs have also been modified for better visualization.

- In the context of the main results presented in this section, Fig 2C and 2D should be removed or moved to Supplementary, since they show results for much later time points (4 hpi and 24 hpi, respectively).

Main text and sections have been re-arranged.

- The final conclusion that "E. faecalis is replicating within the cells" based on the results obtained after 24 hpi per se seems problematic and should be altered, for two reasons:

o Since the experiments were made in fixed different time points, more bacteria might simply have entered in the meantime and persisted inside the keratinocytes.

o In fig Supp 1 the authors show that intracellular bacterial CFU/keratinocyte does not vary significantly from 4 to 24, 48 and 72 hpi with antibiotic treatment (it even decreases in the latter time point).

Please see the various responses above which support the conclusion that *E. faecalis* is replicating within cells.

- It is difficult to assess the significance of the Supp Fig 2, which was provided to show bacteria on the periphery of the keratinocytes that were previously inside the cells (3 hpi + 21 h antibiotic treatment), because these images also do not show any bacteria inside the cells in Z-stack (+ the bacterial amount is very low from what should be expected according to the authors' results). Perhaps invasion did not occur at all in this case? And/or the bacteria outside are due to biofilms that resisted this antibiotic concentration (see above)?

This image is included to show the heterogeneity of bacteria location observed within our infected cell populations. What you are seeing in this figure (now **Supplementary Figure 4**) is not a common sighting, and indeed elsewhere in this well we would find internalized bacteria. The fact that we don't see internalized bacteria in this particular field of view is not surprising given the frequency of intracellularly infected bacteria at this timepoint (<1%). Because these images show *E. faecalis* at the periphery of not particularly healthy looking keratinocytes (lacking clear actin staining, possible with more condensed nuclei), we raise the possibility that they derive from compromised host cells following replication. Of course we cannot prove it at this point. Please see above for our thoughts on biofilm. An equally intriguing possibility is that bacteria that derive from the intracellular niche following replication and/or persistent are not only primed for reinvasion of new mammalian cells (**Figure 8**), but perhaps they are also more tolerant to antibiotics. We have not looked into this yet, but it's an interesting idea.

Entry of E. faecalis into keratinocytes is dependent on actin polymerization and PI3K signalling

- Cytotoxicity assessment of all the compounds tested using dose-response curves and/or imaging should be done (or mentioned, if published previously) to validate the results obtained with keratinocytes, since the authors also detected that at least one (Dynasor) that showed cell toxicity. It is probably important to see if they also affect isolated bacteria (or refer to the literature if they've been shown to have no effect on prokaryotes).

Changed.

- Colchicine seems to boost bacterial adhesion and persistence. Any explanation for this?

When microtubule polymerization is prevented, there is an increase in F-actin polymerization. This effect is widely reported in the cell biology literature. Since we also show that F-actin is important in adhesion and the subsequent internalization, this nicely accounts for the above.

- Are all the mean differences observed In Supp Fig 3 not statistically significantly different (apart from one time with the Dynasore treatment)? If not, the authors should show the statistical analysis for the others as well.

In case they are non-significant, a comment regarding the high heterogeneity of the results obtained should be made (scale is logarithmic).

All significant differences are shown. We do not have an explanation for the heterogeneity observed for some compounds, but we speculate that there may be a degree of stochasticity in the assay in terms of bacterial adherence in particular where the spread is greater.

- The authors cannot completely exclude a role of the endocytocytis (mediated be claveolae and clathrin), based only in the results from Supp Fig. 3, mainly because:

o They have means based on 3 experiments, which in some cases show high heterogeneity between them (so it suggests that in some cases endocytosis via this pathways might occur).

o Authors comment in Supp Fig. 3 that they saw a large amount of Dynasore-treated cells being killed after 4 hpi (justifying that the reduction in intracellular bacteria is due to keratinocytes' death). However, they also saw a statistically significant reduction in intracellular bacteria recovered after 3 hpi (and even with 2 hpi you can see the reduction). This supports the possibility that at least in some cases the bacteria are entering via receptor-mediated endocytosis.

This is a fair point, and we have updated the text to reflect this possibility (~line 160).

o Adhesion of bacteria seems to be impaired with Nystatin (at least in some cases).

It is not statistically significant.

o Controls regarding the abolition of the clathrin and claveolae pathways with the inhibitor concentrations tested were not shown or referred by the authors for this particular cells. As this could vary from cell type to cell type, it's important to confirm that the inhibitors are working as expected.

Please see above, response to editor point 4. Nystatin and Dynasore have been used on HaCaTs and phenotypes measured at the concentrations used on our study.

o The use of Wortmannin per se does not tell us that only macropinocytosis is being influenced; it could also influence phagocytosis, which keratinocytes are also able to do, and even receptor-mediated endocytosis to some extent.

They should either increase their N or try to assess clathrin- and claveolae mediated endocytosis using imaging analysis. Or otherwise consider that receptor-mediated endocytosis pathways might also have a role (at least in some cases).

We have softened the conclusions to indicate that other uptake pathways, in addition to macropinocytosis, may also be at play in both the results (~line 170) and discussion (~line 726).

- Caption from Supp Fig 3 should be reduced and the "discussion" part should be moved to the main text.

Noted. We have now moved it to the results section (~line 170).

<u>Intracellular E. faecalis traffics through early and late endosomes</u>

- No Z-stacks are shown in this section, so how can the authors know for sure that all the bacteria counted was inside? Were these experiments performed in the presence of antibiotics? (If so, dose-response curve should be presented to show the efficacy of the killing, as per above comments)

Here we are not assuming we're counting *all* of the bacteria inside cells. We simply count fluorescent *E. faecalis* and through a combination of visual inspection of Z-stacks and 3D reconstructions and analysis of colocalization of fluorescence markers by histogram analysis, we can say how many individual bacteria associate with each marker of interest. All experiments in this study were performed in the presence of antibiotics. Please see above for antibiotic killing efficacy. Methods for visualization and quantification of internalized bacteria was added in the M&M section.

- Even with prolonged antibiotic treatment, the authors showed previously that E. faecalis is able to escape keratinocytes (Supp Fig 2). How they can confirm that they are not colocalizing bacteria that are outside or between the cells?

As described above, these instances of proposed extracellular bacteria are rare. In the cases where *E. faecalis* was not associated with intracellular markers, we performed visual inspection of 3D projections on Imaris to rule out dubious cases. Additionally, in cases where actin staining was added, actin can be used as a reference to identify intracellular bacteria; moreover proximity to the nucleus and bacteria in the same plane as the nucleus and intracellular proteins, for instance, were also indicative of intracellularity.

- Authors should also clarify if the total N of bacteria/cell compartments counted per condition is supported by a single image (which it seems to be) or different images. More biological replicates seem to be needed.

N has been added.

- Since the total N of bacteria/cell compartments counted vary considerably according to the different labelings, it becomes difficult to compare the percentages indicated by the author. More replicates are needed.

We believe that this has been improved with inclusion of earlier time-points and new data regarding compartment labelling which should clarify what was previously difficult to assess.

- According to the data, the percentage of E. faecalis in LAMP1-positive cells after 24 hpi is lower than the one observed in 4 hpi. Therefore, the authors cannot conclude that the bacteria are replicating inside the endosomes as they state in their final conclusion statement. Shouldn't this value be higher?

Based on our observations, we are proposing that *E. faecalis* has the ability to modulate trafficking markers, at least in a subset of infected cells. By 24 hpi, it's possible that bacteria-containing compartments could have been altered, losing late endosome markers.

- There is not enough evidence about replication in endosomes based on these data. Live cell imaging and/or more cell counting (statistically significant/biological replicates) should be provided.

New data regarding intracellular replication are provided in **Figure 3**. We used BrdU and RADA labelling to identify bacteria in active state of replication.

- The panel in Supp Fig 4 is redundant, since the figures in the upper panel are only in a very slightly different magnification compared with the lower panel (higher difference in magnification or other regions/images should be provided instead).

Good point. Redundant panel now removed (this is now **Supplementary Figure 10)**.

E. faecalis intracellular infection interferes with Rab5 and Rab7 protein levels

- A large initial part of the paragraph (when comparing to other bacteria) should be moved to the Discussion section.

Moved.

- Similar comments from the section above, regarding the absence of Z-stacks and small N used, apply to this section as well.

- Some comparisons from the strains V583 and OG1RF should be better contextualized. Are both of them equally fit in the conditions tested? (Growth curves should be provided) Were they always inoculated at the same MOI? (the MOIs in this section should be mentioned)

As stated above, OG1RF is the most commonly used lab strain for *E. faecalis*, because it is genetically tractable. V583 is the most common VRE *E. faecalis* strain used in the lab and is sufficiently genetically different from OG1RF that is often used to assess strain specificity of a phenomenon, as we do here. We don't propose (and therefore don't comment) that one is more relevant than the other for these studies or this infection model. MOI mentions were improved throughout the text. Both of these strains grow similarly in the cell culture media used in this study, as shown below, but that's not very relevant to our study because all of the experiments in this manuscript are done in the presence of antibiotics so no "growth" in the media is happening. Please see **Supplementary Figure 1** for the killing dynamics of each strain.

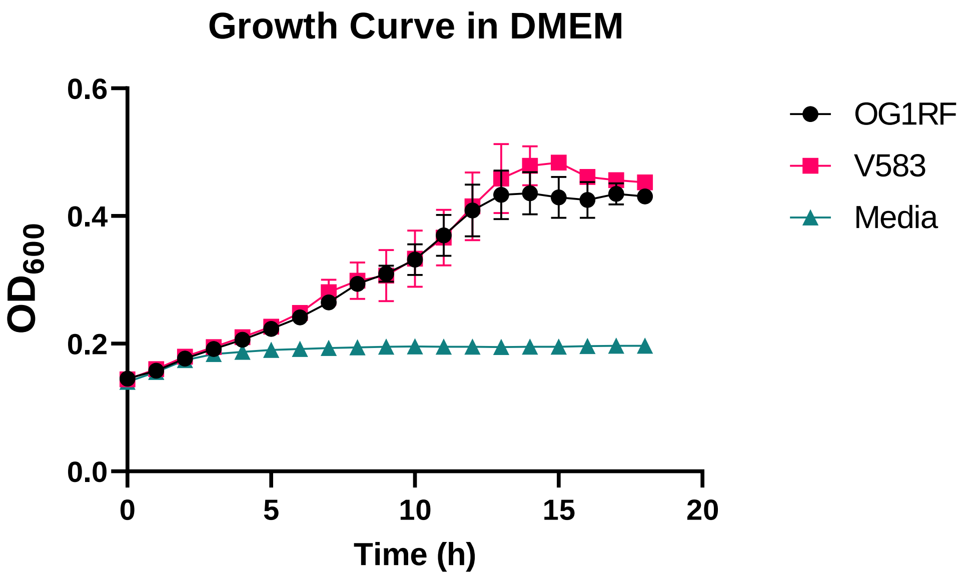

Figure. Growth curve profile of *E. faecalis* strains OG1RF and V583 in DMEM + 10% FBS.

- Since E. faecalis does not change cathepsin D expression (and it seems that it is not naturally reduced over time) and the Rab7 levels seem to be restored after 24 hpi (as the authors also mention), why there is no fusion with lysosomes? This should be better explained or assessed (e.g. targeting/labeling lysosomes) by the authors.

Most compartments lack Rab7 and/or LAMP1 which should therefore fail to fuse with lysosomes. At 24 hpi when Rab7 levels are restored, it's possible that most of the intracellular bacteria in many cells would have completed the trafficking route, avoiding endo-lysosomal labelling. Alternatively, *E. faecalis* could have mechanisms which both reduce Rab7 levels and exclude remaining Rab7 from the endosomal compartments. We elaborate on this in our discussion where we discuss about potential fates *E. faecalis* may encounter when intracellular. Understanding the mechanisms underlying these Rab perturbations are future directions for this project.

<u>E. faecalis containing vacuoles do not fuse with lysosomes</u>

- The authors mention that they did not find evidence of replication, which is quite contradictory regarding the previous results obtained. Could this be related with different MOIs and/or time of infection? In any case, this should be better explained.

These experiments with correlative and light electron microscopy were particularly helpful to help us to understand the nature and heterogeneity of *E. faecalis*-containing compartments, rather than intracellular replication. This is likely a function of the approach, such that we'd have to slice through a replicating cluster at just the right angle to capture it. Nonetheless, we do see hints of replication. For example, in **Figure 7D**, we see two distinct diplococci within a single enclosed membrane-bound compartment. Coupled with the BrdU and RADA staining of intracellular replicating bacteria, these images provide support the conclusion that *E. faecalis* replicates intracellularly.

- Some quantification (with more images/biological samples) is needed and/or experiments targeting lysosomes, in order to conclude the absence of fusion with lysosomes.

Improved with new data on Cathepsin D in **Figure 5 and Supplementary Figure 9**. We now added data for LAMP1 and Cathepsin D, with a monoclonal antibody against LAMP1 (which also labels lysosomes) to identify potential fusion with lysosomes. We were able to observe some instances in which this phenomenon happens.

- In Fig 6 the authors highlight a compartment with "bacteria with altered appearance". This should be better explained in the text. The only lysosome showed in the figure is also not in a commonly observed shape for the compartments.

We agree. Language has been edited.

- Fig 6 and Supp Fig 7 seem to be based in the same (very limited) amount of images.

The main text now states the numbers of intracellular *E. faecalis* analysed, from which these representative images where drawn.

<u>Intracellular E. faecalis is primed for more efficient reinfection</u>

- The authors themselves said that the MOI used for reinfection was very low. This seems quite contradictory, since they also argue that E. faecalis replicates inside the cells over time. Therefore it would be meaningful if a first infection with higher MOIs and/or prolonged time of infection (similar to the ones previously used) was used, in order to recover more bacteria for the reinfection and to correlate better with the rest of the results.

While we might be able to marginally increase the inoculum if we increase the MOI from 50 to 100, MOI above 100 results in cytotoxicity, as does extending the infection time. The important part of this experiment is the comparison of planktonically grown bacteria to those derived from the intracellular niche, where we see the latter is significantly better able to be taken up by keratinocytes. Even though the reinfection experiment was done at lower MOIs, we have no reason to think that increasing the MOI would alter the experimental outcome.

<u>Discussion:</u>

All of the discussion and the E. faecalis persistence model in keratinocytes proposed should be reviewed in light of the aforementioned comments – especially the wound injury focus and in vivo conclusions, the replication of bacteria and the exclusion of receptor-mediated endocytosis in the model. The authors have to somehow account for the fact that there is a reduction over time of the intracellular bacteria recovered in their wound infections in vivo (particularly in CD45- cells), which opposes the idea of replication and persistence of bacteria.

We have edited the discussion as suggestion. Please see response 1b to this reviewer's 1st major comment which explains why intracellular bacteria fall over time despite replication occurring, and how these two findings are not incompatible.

A comment/hypothesis/discussion about how cathepsin D is absent in only 2 out of the 3 possible pathways that they present (and not in all for example) should be made, particularly considering that no change in its cell expression was observed.

Lack of LAMP1/Rab7 will result in lysosomal fusion escape. However, we do see examples in which *E. faecalis*-containing compartments have both labelling indicating that canonical endo-lysosomal pathway happens.

R2 - Summary

In this manuscript, Kline and colleagues detail the intracellular lifestyle of Enterococcus faecalis, a Gram-positive commensal bacterium of the digestive tract. Enterococci are also opportunistic pathogens, colonizing the mouth and wounds. Widely considered as an extracellular pathogen, a number of earlier studies have indicated that E. faecalis can also reside intracellularly. Here the authors use a keratinocyte cell line, HaCaT, as an in vivo model of wound infections. Two strains of E. faecalis, OG1RF and V583, enter these cells, and persist. Bacterial entry is dependent on polymerization of actin cytoskeleton, suggestive of micropinocytosis. Labeling with endocytic markers suggest that maturation of vacuole-residing enterococci follows the endocytic pathway. The correlative and light electron microscopy is a nice addition to the conventional fluorescence microscopy, although the number of bacteria examined is very low. The paper is well-written and easy to follow and the figures are nicely presented. The conclusions are for the most-part justified, with the exception of some concerns that I have detailed below.

Thank you. We appreciate the feedback.

R2 - Major

(1) Figure 2A and 2B. Very few of the total bacterial population are being internalized into keratinocytes (from my estimate comparing Figure 2A with 2B, it is in the order of 1-5%). Is this internalization event an E. faecalis driven event or do keratinocytes have some phagocytic-like activity that allow them to internalize bacteria? Comparing the internalization of E. faecalis with non-pathogenic E. coli (DH10B or K12, for example), or an "extracellular" Gram-positive bacterium, would answer this question.

This is an interesting question, and one we are undertaking in follow-up studies to identify the bacterial effectors that may be involved in the bacterial uptake, intracellular persistence, and intracellular replication in keratinocytes and other cells which may answer this question. Thus, we respectfully propose this is beyond the scope of the current study as the results would not change the conclusion of the present work.

(2) Figure 2C and 2D. Compared to total CFUs (Supp Fig 1), which show a steady-state level of viable intracellular CFUs from 4-48 h, microscopy images show that there is an increase in the number of bacteria/cell with time. This increase cannot be in all infected cells, otherwise there would be an increase in total CFUs. In what percentage of infected cells does this increase in bacterial numbers occur? It is important to know what proportion of keratinocytes support bacteria replication. The authors should count bacteria in individual cells (i.e. single-cell analysis) over a time course to chart the heterogeneity in the

intracellular distribution of bacteria. I suspect there are multiple scenarios happening, with the total CFUs being the sum of (i) bacterial replication in some cells, (ii) no replication in some cells, (iii) bacterial death in some cells and (iv) keratinocyte cell death.

We agree with your suspicion and propose that intracellular replication within a subset of infected cells is not inconsistent with a steady state or even slow decrease in intracellular CFU in the entire population of mammalian cells over time. Particularly in the *in vitro* model in which infected cells supporting intracellular replication are expected to lyse at some point, releasing the intracellular bacteria that will be killed by antibiotics in the media rather than reinfect neighbouring cells. Also, as you suggest, slow decrease in CFU over time could be a net effect of intracellular killing vs intracellular replication -- killing may be happening at a faster rate than the bacteria can replicate. One could imagine that without replication, the intracellular bacteria population would have decreased more rapidly and the extended intracellular persistence reported herein may not have been observed. This hypothesis was in fact proposed by Gentry-Weeks et al (1999) when they showed, for the first time, evidence of extended *E. faecalis* persistence in murine peritoneal macrophages.We do not yet know why we see a slow decline of intracellular bacteria in the *in vivo* model, and this is something we are working hard to understand – how does intracellular replication/persistence *in vivo* contribute to pathogenesis.

In any case, we do not yet have a handle on the precise percentage of infected cells that support replication versus other fates. However, with our newly established the RADA staining assay to definitively assess intracellular replication, we observe 45% (16/35) of the infected HaCaT cells contained intracellular RADA+ *E. faecalis*. We are undertaking a full characterization of the heterogeneity of these intracellular fates in follow-up work. However, qualitative assessments suggest that intracellular replication occurs more readily in macrophages than epithelial cells.

(3) Related to trafficking data and the model depicted in Figure 8. The way that the authors do the infection, it is very asynchronous i.e. with the extended 3 h time period that the bacteria are in contact with the host cells (prior to antibiotic addition) so bacteria will enter almost immediately and others 3 h later. This creates a huge time spread in the trafficking of intracellular bacteria, which complicates the analysis of host cell marker acquisition. I understand that a long infection time is required to increase the number of intracellular bacteria, so I am not faulting the experimental design, but this caveat should be acknowledged. It seems odd to assess EEA1 acquisition at 4 h post-infection when EEA1, an early endosome marker, would only be acquired 15-30 min post-entry. For this reason, it is not surprising that so few bacteria are labelled with this early endosome marker after 4 h. Rab7 is transiently acquired by late endosomes and is lost upon their transition into lysosomes. Its transient nature of association makes it difficult to assess whether a vesicle/phagosome/bacterium has ever acquired Rab7 or not, unless live-cell imaging is undertaken. If a bacterium is negative for Rab7, Rab7 may have been acquired and lost, for example. As depicted in Figure 8, can the authors show that bacterial viability is associated with Rab7/LAMP1-labelling (scenario I, II or III in Figure 8)? Bacteria containing an inducible fluorescent protein could easily be used to assess viability in each of the described vacuoles.

We agree with all of these thoughtful comments. We have added new data looking at *E. faecalis* intracellular trafficking at early timepoints (starting at 30 min) assessing colocalization with the Rab5 marker (**Figures 4 and S7**), where we still see only a subset of

E. faecalis associated with this early endosomal marker. We've also added additional analysis of LAMP1 and cathepsin D colocalization (**Figures 5 and S9**). Similarly, we see a subset of *E. faecalis* that do not associate with Rab7, LAMP1, or cathepsin D, suggesting that at least in some cases, the failure to observe *E. faecalis* in Rab5/7 compartments early on may predict failure to fuse with the lysosome later on, which is consistent with its ability to replicate and persist within a subset of cells. Our response above to the editors summary point 5 also addresses some of these points. Understanding which compartments support *E. faecalis* persistence and replication is, in our opinion, better fitting for a comprehensive follow-up study on this heterogeneity.

(4) The CLEM data requires number of events scored to validate statements such as "all membrane-bound E. faecalis were spatially separated from lysosomes, and there was no indication of membrane fusion between E. faecalis-containing compartments and lysosomes". Specifically, "we observed E. faecalis in LAMP1 positive compartments as well as in vacuoles that appeared to be devoid of LAMP1" please provide percentages; "most internalized bacteria appeared to be morphologically intact", what proportion?; "we did not find evidence of multiple replicating E. faecalis within a single LAMP1 positive compartment leading to membrane distension" how many bacteria were assessed?; "In some instances, however, vacuoles harbouring E. faecalis appeared to contain LAMP1 positive multi-lamellar bodies (MLBs)", how many instances?

The number of events (26 in total) is now stated in the main text (~lines 571-573).

(5) Figure 5B. LAMP1 is a heavily glycosylated protein that migrates at ~100 kDa on SDS-PAGE gels.

We appreciate this observation. In our previous submission, there was an error and the 140 kDa labelling was out of place. The band of interest is between 100 kDa and 140 kDa. As LAMP1 size can vary from 90-120kDa, we believe the band shown corresponds to the correct size range. This has been confirmed when we used the suggested monoclonal antibody (**Supplementaty Figure 11**).

The image shown in Figure 5B suggests that the LAMP1 antibody used in this study is not actually specific for LAMP1. Monoclonal H4A3 (available from Developmental Studies Hybridoma Bank) and D4O1S (Cell Signaling, https://ddec1-0-en-ctp.trendmicro.com:443/wis/clicktime/v1/query?url=https%3a%2f%2fwww.cellsignal.com%2fproducts%2fprimary%2dantibodies%2flamp1%2dd4o1s%2dmouse%2dmab%2f15665&umid=45f6c509-1b95-4c4b-afc9-99a51ac4e759&auth=d0d2ee656b1a8c5d3181c6674d023d4ed062f583-ba90c7f06f847d495704f85f1c5d6a37ccd58083) are validated antibodies for LAMP1. I would like to see that the LAMP1 data is reproducible for either of these antibodies. Given that the authors are drawing conclusions about the steady-state levels of endocytic proteins, it is imperative that they are actually detecting LAMP1 here.

Thank you for this suggestion. We bought the antibody, and new LAMP1 data with monoclonal antibody was added, which confirms our previous findings (**Figures 5B,C and Supplementary Figure 9 and 11**).

R2 – Minor

(1) The source/description of E. faecalis V583 is not listed in Materials and Methods.

This has been added.

(2) Figure 5C. Text size is too small to be easily seen.

This has been amended.

(4) Supplementary Figure 1B and C. What is the dashed line?

This has been changed in the text. The dashed lines simply serve as a reference for 10^4 CFUs. Since the intial CFUs vary between the experiments performed for OG1RF and V583, the reader can easily visualize that V583 CFU are higher at early time points.

(5) Fluorescence images. As suggested by a number of journal commentaries (https://ddec1-0-en-ctp.trendmicro.com:443/wis/clicktime/v1/query?url=https%3a%2f%2fpubmed.ncbi.nlm.nih.gov%2f22379119%2f%3b&umid=45f6c509-1b95-4c4b-afc9-99a51ac4e759&auth=d0d2ee656b1a8c5d3181c6674d023d4ed062f583-6517073a101b2bfb5569bb0a3b7d9e6b0082c2bd https://ddec1-0-en-ctp.trendmicro.com:443/wis/clicktime/v1/query?url=https%3a%2f%2fwww.ascb.org%2fscience%2dnews%2fhow%2dto%2dmake%2dscientific%2dfigures%2daccessible%2dto%2dreaders%2dwith%2dcolor%2dblindness%2f&umid=45f6c509-1b95-4c4b-afc9-99a51ac4e759&auth=d0d2ee656b1a8c5d3181c6674d023d4ed062f583-bd2e33ffa076f0364959f57c0a749bb449fd11f0), fluorescence images should be shown in greyscale, where possible, to improve visibility to the human eye and accessibility to those with color blindness. This impacts Figure 4, Supp. Fig 4, Figure 5, Supp. Fig 5.

We appreciate this comment and will take this on board in our follow-up studies. In instances where compartment labelling was shown, we hope that *E. faecalis*-containing compartments can be visualized independently of the colours used. We also provide intensity profile histograms that may clear any possible doubt about colocalization in some other images. As an aside, and appreciating that color blindness can manifest differently in different people, two of the authors on this manuscript are color blind and did not have a problem with our color choices; hence, this fell off our radar.

R3 - Summary

The authors describe the ability for E. faecalis to invade immune and non-immune (namely keratinocytes) cells during a murine wound infection model. This phenomenon, as with other sites of the body, appears to confer significant biological advantages for the microbe, allowing it to persist in spite of a robust immune response and potentially traditional antimicrobial treatments. The authors performed an extensive and well selected set of experiments in order to begin unravelling the mechanisms of invasion. This manuscript is an

interesting, important and well written piece of work. However, there are a number of minor issues that I believe require further explanation and clarification before publication.

R3 - Major

If possible the authors should present the gentamicin protection assay final wash CFU's. The addition of this data would significantly improve this manuscript

Great suggestion. We now provide this additional data (**Supplementary Figure 1**) and observe only a minute fraction of the intracellular population can be recovered in the final PBS wash, likely indicative of sloughed infected cells.

R3 – Minor

“whereupon it manipulates the endosomal pathway and expression of trafficking molecules required for endo-lysosomal fusion, enabling E. faecalis to avoid lysosomal degradation and consequent death.”

Qualify language – data does not necessarily prove that the bacteria manipulates the endosomal pathway “extracellular pathogen”

Is it still thought of as an extracellular pathogen? Please consider rephrasing

We have added substantial new data to this revision demonstrating that *E. faecalis* undergoes heterotypic intracellular trafficking. In one subpopulation it appears to traffic “normally” in association with the expected stage specific markers in the endo-lysosomal pathway, whereas another subpopulation fails to associate with any of the canonical pathway markers and which may explain why some persisting intracellular *E. faecalis* appear never to associate with lysosomal markers. Further, we add additional data showing that infection with *E. faecalis* suppresses Rab5 and Rab7 protein levels, consistent with a model in which *E. faecalis* infection results in manipulation or modulation of this pathway. Nonetheless, we’ve softened the language to say instead “Here, we report that E. faecalis become internalized into keratinocytes primarily via macropinocytosis, whereupon they undergo heterotypic trafficking through the endosomal, which enables their replication and survival”.

As for whether *E. faecalis* is still an extracellular pathogen – a resounding yes! Based on our wound infection data, we can estimate that approximately 1-10% of the total recovered bacterial population are intracellular at these time points and the rest are extracellular. We comment on this in the first section of the results.

Results

Intracellular E. faecalis are present within CD45+ and CD45- cells during mouse wound Infection

Were the gentamicin treated “extracellular” bacteria definitely dead (live/dead stain)? Was this strain gent susceptible? In our experience, this assay can be quite misleading – did you plate the final PBS washing steps on agar to ensure no/low growth?

Please see also above, response to editor point 1, and our response to this reviewer’s major comment above.

Fig 1. Are you plotting biological or technical replicates? You mention that at least 3 replicates were used. Would it be possible to clarify this in the legend?

This text has been amended to reflect that we are plotting biological replicates.

E. faecalis adheres to and enters keratinocytes

Again, the results from the gentamicin protection assay can be very misleading. If you have no cfu data from the PBS washes, then perhaps qualify the language.

Again, please see also above, response to editor point 1, and our response to major comment above.

Nonetheless, the imaging data is compelling and beautifully presented. Perhaps make the orthogonal lines a little clearer for the reader.

Thank you, and we have made thicker the orthogonal lines.

Supplementary Fig 2. Convincing images but please increase magnification

We appreciate this comment, because we wanted to show a better perspective of surrounding cells, we provide an increased in size image now and hope it can be better visualized now.

Entry of E. faecalis into keratinocytes is dependent on actin polymerization and PI3K

Signalling

Fig. 3 and Supplementary fig. 3. This data is convincing; however, the statistical significance is less so. Are all data points technical or biological repeats? The use of parametric tests for such small datasets is unusual. Was the data normally distributed? If not consider non-parametric tests. Indeed, it might be better to not do tests - the difference can be seen in the graphs

The data reflect biological replicates, each of which comprises an average of a minimum of three technical replicates.

Intracellular E. faecalis traffics through early and late endosomes

How was this percentage data calculated? Was this done using image analysis software or done manually? This needs some explanation. If analysed manually, then how many cells / experiments were included. The images (particularly the red channel) look to have been increased in brightness.

We count fluorescent *E. faecalis* through a combination of visual inspection of Z-stacks and 3D reconstructions and analysis of colocalization of fluorescence markers by histogram analysis, we can say how many individual bacteria associate with each marker of interest. We included information on how data was analysed in the material and methods.

E. faecalis intracellular infection interferes with Rab5 and Rab7 protein levels

"For instance, Mycobacterium tuberculosis affects Rab7 recruitment and, consequently, phagosome maturation, by interfering with Rab5 effectors (Saikolappan et al. 2012; Puri, Reddy, and Tyagi 2013). Listeria monocytogenes also affects Rab7 recruitment by inhibiting Rab5 GDP exchange activity in host cells (Prada- Delgado et al. 2005). Additionally, Coxiella burnetii can localize to Rab5 and LAMP1 positive compartments that lacks Rab7 (Ghigo et al. 2009; Ghigo, Colombo, and Heinzen 2012)."

I wonder whether this above section should be moved to the introduction / discussion. Or perhaps shortened. It feels out of place.

We appreciate this comment and have moved this to Introduction.

As mentioned earlier, it is not clear how colocalization with intracellular compartments was measured. Percentages are stated but the method is not clear. Please add. Without this information it is quite difficult to judge how reliable this data is.

As per the answer above, we counted fluorescent *E. faecalis* through a combination of visual inspection of Z-stacks and 3D reconstructions and analysis of colocalization of fluorescence markers by histogram analysis, we can say how many individual bacteria associate with each marker of interest.

Is the WB data normally distributed? T-test is parametric

Normality tests were performed, and normality confirmed, before proceeding with other statistical analyses.

E. faecalis containing vacuoles do not fuse with lysosomes

Fig. 6 and supplementary Fig 7. CLEM is very nicely presented and looks convincing. It might be easier for the reader to mention the colours in the legend. It is currently quite difficult to understand

Thank you for the kind compliment. We have amended the legend as suggested.

Intracellular E. faecalis is primed for more efficient reinfection

“These results are similar to observations made in S. pyogenes, where longer periods of internalization in macrophages increased recovered CFU during subsequent reinfections (Hertzen et al. 2012).”

The above sections belongs in the discussion

Fig 7. (A) I agree with the use of non-parametric tests for these experiments. However, why did you not use this for the previous tests? The N appears to be comparable….

We avoided non-parametric tests where the number of biological replicates are small (eg. N=3) as per Graphpad guidelines (https://www.graphpad.com/guides/prism/latest/statistics/when_to_choose_a_nonparametric.htm). It seems that choosing one method or another is conflictive in literature for small size samples (https://doi.org/10.1002/sim.7263), but as per Graphpad guidelines, which we follow throughout the paper: nonparametric tests have little or no power to find a significant difference in small size sample. In some instances where we did use parametric tests, normality was first assessed and verified.

Discussion

“This is well described for uropathogenic E. coli”

This statement is true. However, this is in murine models of infection. Very little data in humans. Please alter language.

We have amended the sentence as follows: This is well-described for uropathogenic *E. coli* (UPEC), particularly in animal models in which UPEC can replicate to high numbers within urothelial cells as intracellular bacterial communities or can persist in a quiescent intracellular state within LAMP1+ compartments for long periods of time, promoting recurrent and chronic infection (44-47).

I think it is important to clarify that this a mouse / cell model of infection. Experiments using infected wounds in humans would need to be performed to corroborate this data.

We have amended the subsequent discussion sentence as follows: Here we report that, *in vitro*, *E. faecalis* become internalized into keratinocytes via macropinocytosis, whereupon they manipulate the endocytic-lysosomal pathway, enabling their replication and survival.
