# Supplementary material for: Enterococcus faecalis alters endo-lysosomal trafficking to replicate and persist within mammalian cells
Source: PLoS Pathog. 2022 Apr 7;18(4):e1010434. doi: 10.1371/journal.ppat.1010434 (PMC9017951; doi:10.1371/journal.ppat.1010434)
Supplement: Attachment — Submitted filename: Response to reviewers #2_05032022.pdf [file ppat.1010434.s018.pdf]

After assessing your responses to the Reviewers' comments and the accompanying revisions and additions incorporated into the revised manuscript, we find that the revised manuscript represents a major improvement that in all major respects addresses the reviewers' concerns satisfactorily. There are, however, some minor issues remaining, primarily associated with the novel information included, that could be easily amended, and that would not require any additional experiments. These issues are listed below. As long as these minor revisions and amendments to the current manuscript are done, we would expect to return a favorable decision regarding your manuscript very quickly.

We are pleased to read this and happy you share the same excitement about our paper and findings.

1. Based on the request from the reviewers, additional imaging data has been included to better represent the data and their associated conclusions (Fig 4A-D, 5B-C, S7, S8, S9). However, Figures S7 and S9 have no arrows in the individual panels making it difficult to understand what should be observed. Please add.

Arrows to the individual panels have now been added.

New Figures 4A-D and 5B-C are relevant additions, yet it is hard to see the staining and co-positioning/co-localization with variable staining intensity of blue, red and green colors and no separate panels for each channel.

We appreciate that this can be difficult to see, which is why we added the separated panels of the representative examples in the supplementary figures (Figs S7 and S9). We hope that the reader can access the support information to evaluate the individual panels for themselves.

The histogram analysis it a welcome addition, although without the ability for the reader to visually evaluate co-localization or co-positioning of bacteria and markers, the specific criteria used to score this, as described in the Methods section (lines 983-988) becomes unclear and difficult to follow. For example, in Figure 5B-C, the 4 h time-point, why is the first bacteria in the histogram not considered co-localized with LAMP-1 and in the 24 h section, again why is the first bacterium not considered co-localized with LAMP-1? Is this based on signal intensity or visual information that is not apparent unless each marker is observed separately? Additional clarification of the co-localization/co-positioning analyses in these figures would be necessary.

We scored the labelling of each *E. faecalis*-containing compartment using the signal intensity with subsequent visual inspection to verify the accuracy of the scoring. To make this clearer, the following sentence had been incorporated in the figure legends for the reader's reference: (~lines 324-326; 381-383) "Labeling of individual cells was manually scored using a combination of histogram overlap (where each cell is a peak on the histogram) and visualization of orthogonal views and 3D projections generated using Imaris 9.0.2."

In addition, where clear label overlap was observed in the histograms, we added arrows to show representative examples of co-localization that, again was further verified with visual inspection. By this criterium, the first bacteria in question at the 4 h time point is co-localizing with LAMP1 (we have now added another black arrow to show that too). At 24 h, LAMP1 is nearby but it does not coincide with the localization of the bacterium, so we did not consider that example as co-localized. We also added the following sentence to the main text so the way we analysed *E. faecalis*-containing compartments is clear: (~lines 279-281) Fluorescence histograms were generated for individual *E. faecalis*-containing compartments and were further validated by visualization of orthogonal views and 3D projections.

2. A new Figure 3C-D has been added to directly address intracellular replication by showing BrdU and RADA staining of bacteria inside HaCaT cells as well as in RAW macrophages. For both cell types in Figure 3C, arrows would be needed also in the separate panels to orient the reader to the colocalization events.

Arrows have now been added.

The RADA staining for the HaCaT cells shows unspecific and dotty staining where no bacteria are found. Please comment or explain.

We believe that the dotty staining is a non-specific artifact of the HaCaT staining process since there was no bacterial signal near those areas. Although most of the unincorporated RADA are washed out of the bacteria after permeabilization and PBS washes, it seems that with HaCaT cells RADA is "stickier" than with macrophages, for unknown reasons. We are confident that bacteria incorporated RADA, first because macrophages don't have the same issue, and second because of the proper shape and signal that coincides between bacteria and RADA.

3. Reviewer 1 and 2 indicated a lack of information about endosomal trafficking inhibitor function. Inhibitor function is indirectly addressed in the response to Reviewer 1 through a list of references of papers that have used the same concentrations in the same cell types by other investigators. This information is, however, not included in the manuscript and the use of concentrations and inhibitors listed on lines 913-915 in the Methods section is not complete. To justify the inhibitor concentrations used in the manuscript, in the absence of experimental validation, please include the specific concentrations used for each inhibitor in the Methods section together with the references used to justify these concentrations.

We have now added this information (~lines738-752).

4. Reviewer 2 highlights that the LAMP-1 antibody used may not be optimal and suggests validated antibodies to be used for confirmation. One of the validated

antibodies suggested is now used in new figures Fig 5, S9, S11. However, although this is commented on in the response to the Reviewer, it is not mentioned specifically in the manuscript when different antibodies were used. As validation using these antibodies was an issue, it would be relevant to include antibody information for the LAMP-1 staining either in the relevant figure legends or mention in the Methods section what LAMP-1 antibody was used for what Figure, so that the readers can properly interpret the data.

We have indicated whether the poly or monoclonal LAMP1 antibody was used in the relevant figure legends. Where we state the antibody used, we put between parentheses polyclonal or monoclonal antibody (eg.: Fig5 and Fig6). However, to make this clear and to avoid doubt, we have now added the following sentences to the material and methods: (~line 779-781) "LAMP1 was visualized using polyclonal mouse α-LAMP1 (ab25630, Abcam, Cambridge, UK) or monoclonal mouse mAb α-LAMP1 (D4O1S, Cell Signalling Technology, USA), as indicated in the figure legends".
